# Molecular assessment of drug-phospholipid interactions consequent to cancer treatment: a study of anthracycline-induced cardiotoxicity

**DOI:** 10.1038/s41598-023-48184-4

**Published:** 2023-12-13

**Authors:** Yara Ahmed, Khalil I. Elkhodary, Mostafa Youssef

**Affiliations:** 1 Nanotechnology Program, The American University in Cairo, AUC Avenue, P.O. Box 74, New Cairo, 11835 Egypt; 2 Department of Mechanical Engineering, The American University in Cairo, AUC Avenue, P.O. Box 74, New Cairo, 11835 Egypt

**Keywords:** Computational biology and bioinformatics, Computational biophysics

## Abstract

Cardiotoxicity limits the use of anthracyclines as potent chemotherapeutics. We employ classical molecular dynamics to explore anthracycline interactions with a realistic myocardial membrane and compare to an ideal membrane widely used in literature. The interaction of these two membranes with four anthracyclines; doxorubicin, epirubicin, daunorubicin, and idarubicin are studied. Careful analysis was conducted on three forms of each drug; pristine, primary metabolite, and cationic salt. By examining the molecular residence time near the membrane’s surface, the average number of molecule/membrane hydrogen bonds, the immobilization of the molecules near the membrane, and the location of those molecules relative to the mid-plane of the membrane we found out that salt forms exhibit the highest cardiotoxic probability, followed by the metabolites and pristine forms. Additionally, all forms have more affinity to the upper layer of the realistic myocardial membrane. Meanwhile, an ideal membrane consisting of a single type of phospholipids is not capable of capturing the specific interactions of each drug form. These findings confirm that cardiotoxic mechanisms are membrane-layer and drug-form dependent.

## Introduction

Anthracyclines are a widely used class of anticancer drugs owing to their clinical efficacy in treating a wide range of malignancies in both children and adults^1^. Their cytostatic action is mediated by inhibiting DNA synthesis through intercalating nucleic acids and/or inhibiting Topoisomerase II^2^. The most common detrimental side effect presented by anthracyclines is cardiotoxicity^1,2^. Unfortunately, the mechanism by which anthracyclines damage the heart cells remains elusive to date^1,2^. Experimental and clinical studies confirmed that the anti-tumor action of the drugs is independent and separable from their cardiotoxic activity^3,4^. During the treatment period, reversible cardiac impairment symptoms may manifest. This is known as acute toxicity^2,3^. In contrast, more severe, irreversible cardiac issues may present within a year: early chronic cardiotoxicity, or several years after the anthracycline chemotherapy has stopped: late chronic cardiotoxicity, common in survivors of childhood cancer^2,3,5^. Hypotheses explaining anthracycline-induced cardiotoxicity include generating reactive oxygen species, altering iron and/or calcium homeostasis, mitochondrial dysfunction, impaired gene and protein expression, and direct interactions with the myocardial membrane^6,7^. Regardless of the mechanism of cardiotoxicity, a key step in initiating cardiotoxic action is an anchoring of the molecule to the membrane’s surface, where it either crosses the myocardial membrane into the heart cell or is stored within the bilayer itself^8–10^.

The four most popular molecules of this class are doxorubicin (DOX), epirubicin (EPI, the stereoisomer of DOX), daunorubicin (DAU), and idarubicin (IDA). These forms will be referred to as the pristine forms of the drugs in this study. At equal concentrations, EPI exhibits less cardiac toxicity than DOX. However, its risk of cardiotoxicity is not eliminated^4,11,12^. In fact, none of the pristine molecules exhibits significant cardiac tolerability relative to the others^4,11,12^. To be solubilized for injection into a patient, these four lipophilic anthracycline molecules are converted to a salt protonated form by reacting with an acid^13^. The most available anthracycline prescriptions are hydrochloric acid salts: DOXH^+^, EPIH^+^, DAUH^+^, and IDAH^+^^14–18^. Following drug intake, anthracyclines are metabolized by hydroxylating the carbonyl group on carbon number 13 into a hydroxyl group^3,4^. Although this metabolism mainly takes place in the liver, the enzymes responsible for this conversion are also present in cardiomyocytes and are able to produce metabolites intracellularly^3,4^. The major metabolic pathway of anthracyclines produces secondary alcohol forms, viz. doxorubicinol (DOXol), epirubicinol (EPIol), daunorubicinol (DAUol), and idarubicinol (IDAol). The molecular structures of all 12 forms are illustrated in Supplementary Fig. S1.

As noted earlier, the seed step for the cardiotoxicity of these different forms of anthracyclines associates with their anchoring to the upper (outer) surface of the myocardial membrane as a key process. Following this step, the molecules can cross the bilayer structure of the myocardial membrane into the cell to induce further cardiotoxic actions. The crossing is known to take place via a simple diffusion process across the myocardial membrane^14,19^. Alternatively, the anthracycline molecules may remain trapped within or near the lipid core of the two layers of the myocardial membrane^8^. In this computational study we only focus on the key aspect of surface-anchorage to rank the relative cardiotoxic “potentials” of these different forms of the anthracyclines. We simulate their interactions with the myocardial membrane using classical molecular dynamics (MD). To assess this molecular anchorage, we consider a set of parameters. These include, the residence time of the molecules in the neighborhood of the bilayer structure, their proximity to the mid-plane parallel to the bilayer structure, the formation of hydrogen bonds between the molecules and the phospholipids of the membrane, and the ability of the membrane’s surface to immobilize or slow down these molecules.

There have been prior attempts to computationally assess the interactions between anthracycline molecules and membrane structures^6,7,20,21^. Therein, the focus was not necessarily directed towards cardiotoxicity nor captured the essential features of the myocardial membrane^6,7,20,21^. Indeed, the membrane models used in past studies were made up of only one^20,21^ or two phospholipids, and some included cholesterol^6,7^. Those assemblies of phospholipids provide insight into anthracycline-membrane interaction in general, not specific to the myocardial membrane. Moreover, they do not capture the true complexity of the myocardial membrane^22^. With respect to the different molecular forms of anthracyclines that would exist inside the body, a previous paper focused on the pristine forms but not the salt or major metabolite forms of the molecules^20^. Some other studies focused on DOX only^6,7^, while a more recent study explored the behavior of quinone moiety specifically^21^. Our study is more expansive in its assessment of 12 anthracycline forms, and highlights the necessity for a realistic myocardial membrane model to understand the dynamics and interactions that could be the potential cause of the cardiotoxic effect of anthracyclines.

## Results

### Section 1: A realistic myocardial membrane model

**Figure 1:**
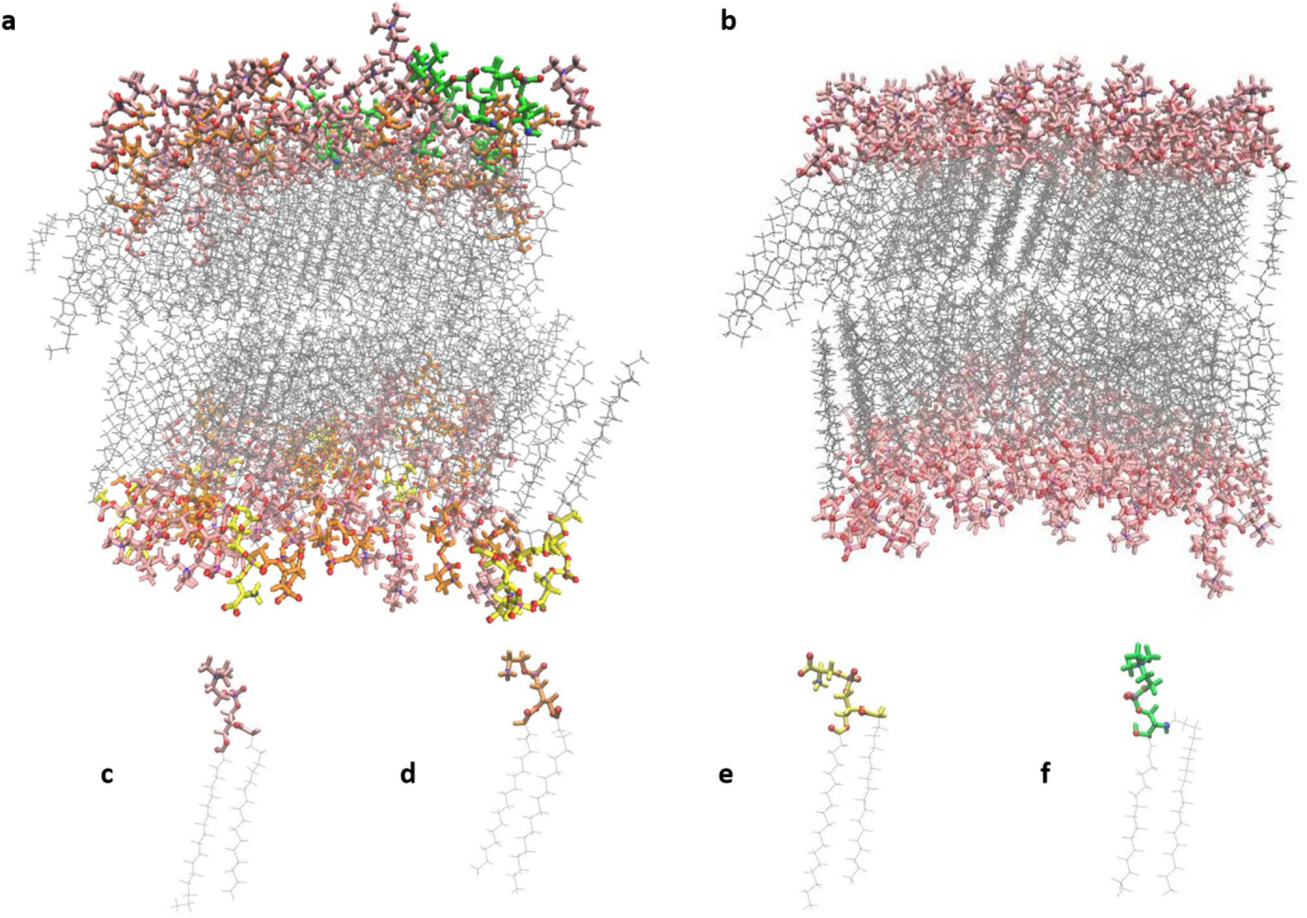
Myocardial membrane model versus ideal DSPC membrane model. (**a**) Myocardial membrane made out of 4 types of phospholipids, (**b**) Ideal DSPC membrane, (**c**) Zwitterion 1,2-Distearoyl-glycero-3-phosphatidylcholine (DSPC), (**d**) Zwitterion 1,2-Distearoyl-glycero-3-phosphatidylethanolamine (DSPE), (**e**) Negatively-charged 1,2-Distearoyl-glycero-3-phosphatidylserine (DSPS) and, (**f**) Zwitterion Stearoylsphingomyelin (SSM). Water molecules and sodium chloride ions are not shown here for clarity.

We focus on the unique organization of phospholipids that comprise the myocardial bilayer as their arrangement will affect anthracycline anchorage, trapping, and penetration^14,19^. In this study, several membrane models were tested following the experimental findings of the lipidomic study of Matos et al.^22^. The most suitable candidate for our study was selected based on the membrane model’s stability, fluidity, and resemblance to the experimentally reported myocardial cell membrane composition. The tested factors are the membrane’s temperature, number of phospholipids, hydration ratio, the salt concentration in the hydrating water, and membrane phase. Figure 1a shows the final representative model chosen, which contains 64 phospholipids per layer. We compare our model to a simpler membrane model prevalent in the literature^23^ that comprises one type of phospholipid, as shown in Fig. 1b. Additionally, the four phospholipids in the two membrane systems are presented in Fig. 1c–f. The details of the myocardial membrane model’s composition are listed in Table 1 and compared to experimental reports^22^. We note that the slight differences between the phospholipid percentages in our computational model and the experimental ones are due to the necessity of matching the surface areas of the lower and upper layers in the computational model and due to the absence of cholesterol in our representation. Another reason is that we simplify the composition of the lipid tails to render our model tractable. As shown in both Fig. 1 and Table 1, the myocardial membrane is asymmetric in terms of phospholipids distribution among the two layers, with SSM (Stearoylsphingomyelin ) present only in the upper layer and DSPS (1,2-Distearoyl-glycero-3-phosphatidylserine), a negatively charged phospholipid, exclusive to the lower layer. All lipid tails were kept constant, with saturated chains of 18 carbons, except for one of the SSM tails, which includes a double bond between carbons 4 and 5, indicated in Fig. 1f as a branched chain. The choice of the length and saturation of the lipid tails was made based on the most common lipid chain present in the myocardial membrane as reported by Matos et al.^22^ As for SSM, the lipid tails of this phospholipid naturally exist in non-matching lengths and saturations, as a result, the second most common lipid tail type for this head group was chosen. The membrane’s temperature is set to 310 K and the salt concentration is 0.15 M, mimicking bodily conditions. The phospholipids are sufficiently hydrated by water molecules with a hydration ratio of up to 50:1, or slightly less.

**Table 1:** Lipidomic details of the myocardial membrane model adopted in this study versus the actual lipidomic findings determined experimentally in reference^22^.

Phospholipid head group	% (and number) of each head group in our membrane model		Experimental results of the head group composition as reported by Matos et al.^22^	
	Upper layer	Lower layer	Upper layer	Lower layer
Phosphatidylcholine	62.5% (40)	54.5% (35)	52%	52%
Phosphatidylethanolamine	26.5% (17)	33% (21)	24.95%	24.95%
Phosphatidylserine	0%	12.5% (8)	0%	7.2%
Sphingomyelin	11% (7)	0%	13.5%	0%
Phospholipid head group	Lipid tails distribution in our membrane model		Experimental results of the lipid tails distribution as reported by Matos et al.^22^	
Phosphatidylcholine	PC, PE and PS are all paired with 18:0/18:0 lipid tails i.e. Both lipid attachments are saturated, 18-carbon-long chains		The most common lipid chain among the head groups is the 18 carbons-long, saturated chain 18:0 The second most common is the unsaturated 20:4 chain. It contains 20 carbons and 4 double bonds The third is 16:0	
Phosphatidylethanolamine				
Phosphatidylserine				
Sphingomyelin	SSM is the only phospholipid whose head group is paired with 18:1/18:0 lipid tails. One lipid chain contains one double bond between carbons 4 and 5 and the other is saturated. Both chains are 18-carbons-long			

### Section 2: Anthracycline interactions with the myocardial membrane

The resulting myocardial membrane is simulated in a drug-free environment for benchmarking. Likewise, each drug molecule is simulated alone in a bulk water environment. These benchmarks are followed by simulations that introduce a single drug molecule to our myocardial membrane, totaling an additional 12 simulations. Then, these 12 simulations are repeated, but replacing our myocardial membrane model with the simple DSPC (1,2-Distearoyl-glycero-3-phosphatidylcholine) membrane for comparison. Computational details are outlined in the “Methods” section. Details concerning the thermodynamic stability of the myocardial membrane are presented in Supplementary Section S1, Figures S2 and S3, and Table S1.

Consistent with Yacoub et al.^6^ and experimentally reported observations^19^, we do not observe any of the drug molecules completely crossing the myocardial membrane under unbiased MD simulation conditions. This is unlike the report of Toroz and Gould^20^, however, which we deem inconsistent with the confirmed time scale of minutes required to observe an unbiased crossing event^19^. Crossing phenomenon require the order of minutes to overcome the known high-energy barriers of this event^6,19^. Some form of accelerated dynamics could be however employed to visualize the translocation of the drug molecules across the myocardial bilayer within feasible computation time. This should be considered on future work focusing on the crossing kinetics. Under our simulation conditions, only two molecules, EPIH^+^ and DAUH^+^, have succeeded in partially permeating the upper layer of the myocardial membrane (see Supplementary Movies S1 and S2, respectively).

#### Residence time

**Figure 2:**
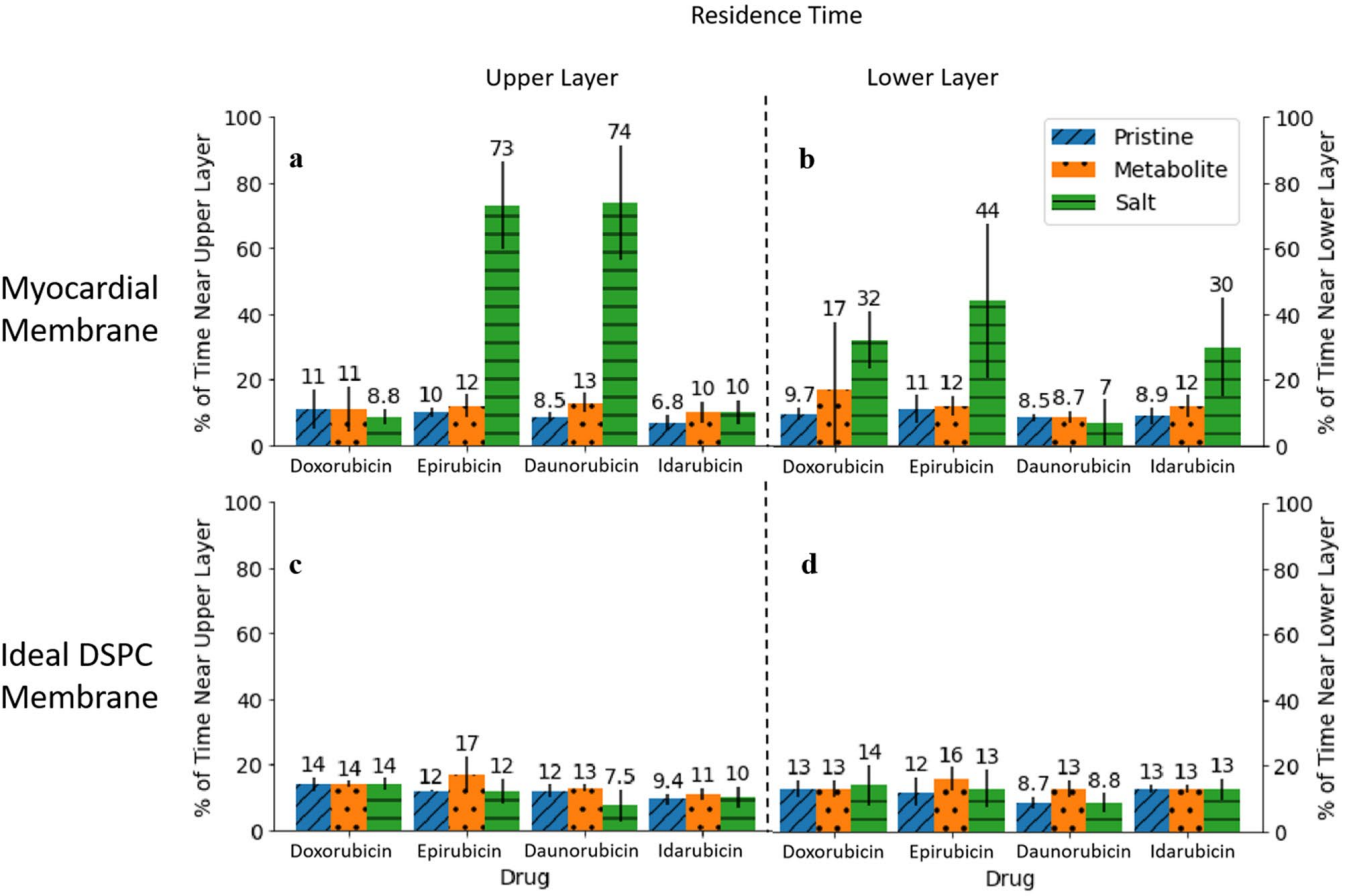
Residence time of each anthracycline molecule near the myocardial membrane’s surfaces and the ideal DSPC membrane’s surfaces. Percentage of time spent near (**a**) the upper layer of myocardial membrane, (**b**) the lower layer of myocardial membrane, (**c**) the upper layer of ideal DSPC membrane, and (**d**) the lower layer of ideal DSPC membrane. The error bars represent the standard deviation which is calculated by dividing last 800 ns of the trajectory into 4 blocks.

We calculated the residence time as the percentage of the simulation time the molecule spends at a proximity of 0.3 nm or less near the membrane’s surfaces; upper and lower layers. This 0.3 nm is selected as it represents the average length of a biological salt bridge^24^. It is worth noting that the distance between the molecule and a surface of the bilayer structure is calculated as the shortest distance between an atom in the molecule and an atom on the surface. As an example, if a molecule spent a total of 0.6 µs at a distance that is less than or equal to 0.3 nm from the lower layer, and the total simulation time is 1 µs, then the residence time of the molecule near the lower layer is calculated as follows: $\frac{0.6}{1} \times 100 = 60\%$. Figure 2 illustrates the residence time of each simulated molecule near the myocardial membrane and the simple DSPC membrane.

The first general trend observed here is that the simple DSPC membrane does not accurately portray the molecules’ behavior, especially the salts. All molecules' residence times seem uniform for this idealized membrane. However, these residence times actually vary widely, as revealed by our myocardial membrane. Specifically, the residence times of all molecules for the ideal DSPC range from 7 to 17% of the simulation time. However, salts tarry near our myocardial membrane for substantially longer times (multiples of times those of the ideal model). This finding is especially interesting when we look at the residence time near each individual phospholipid in our myocardial membrane (Supplementary Table S2). We find that all molecules spend the longest residence time near the DSPC of our myocardial membrane. That is, molecules prefer to spend more time near our myocardial DSPC than that of the idealized model. This finding highlights a cooperative effect of neighboring phospholipids of different head groups^25^ that is missed in the idealized model. For our myocardial membrane, however, neighbors do contribute to the stabilization and residency of anthracycline molecules near the DSPC phospholipid.

We next compare the residence times of the different salts in relation to the upper and lower layers of the membrane (asymmetric). EPIH^+^ shows the longest residency near both layers of our myocardial membrane. It spends 73% and 44% of the simulation time near the upper layer and lower layer, respectively. In fact, we find that it often falls well within the myocardial bilayer (as can be seen in Supplementary Movie S1), becoming simultaneously near both layers. DAUH^+^ is the second molecule in terms of residency, spending 74% of the time near the myocardial upper layer but only spending 7% near the lower layer. Again, it partially permeates the membrane but not to the extent of being simultaneously close to both the upper and lower layers as in the case of EPIH^+^. Conversely, DOXH^+^ and IDAH^+^ prefer to reside near the lower layer of the myocardial membrane. One more noteworthy trend is that of the metabolites. They exhibit residence times that are longer than or equal to the pristine forms.

#### Hydrogen bonds

**Figure 3:**
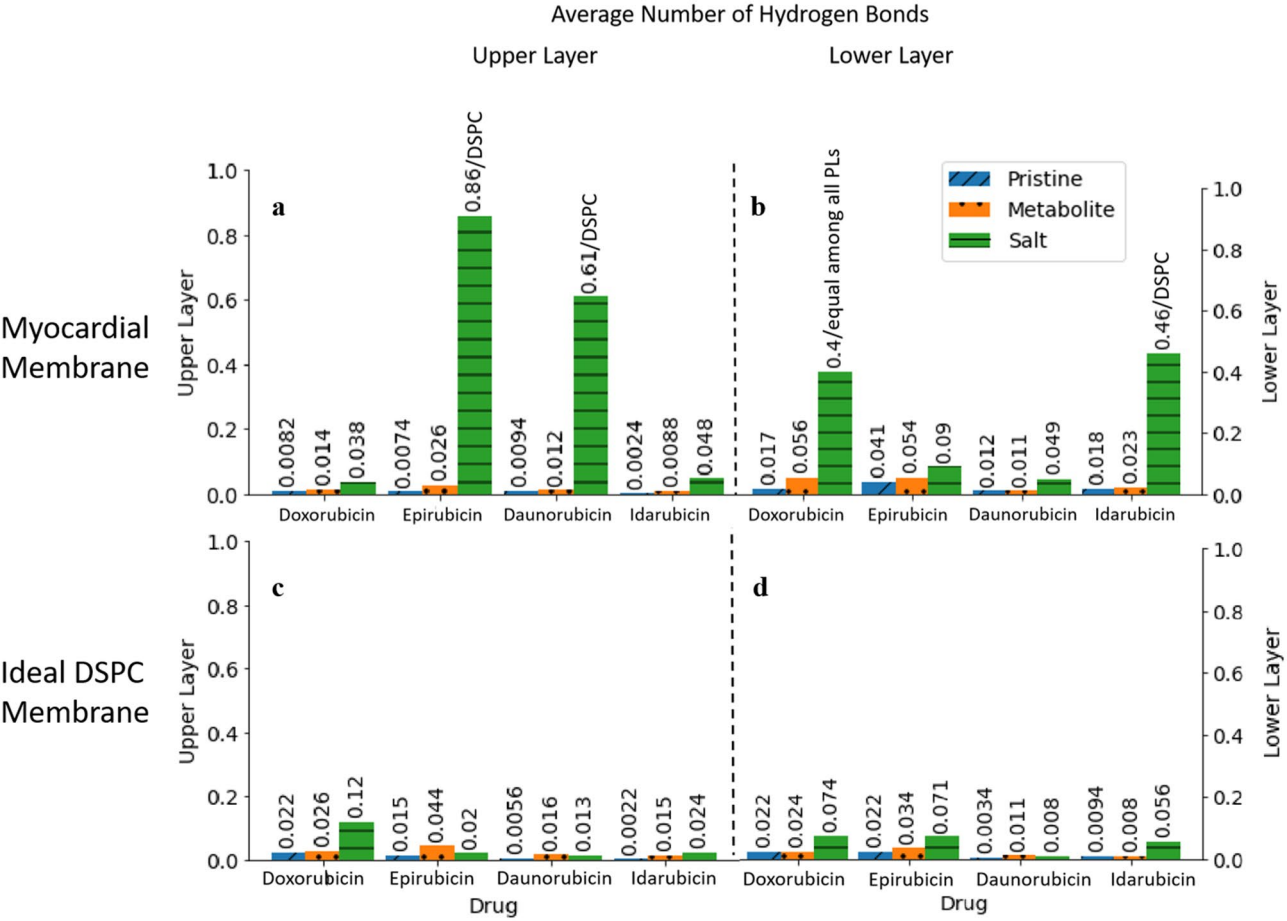
The average number of hydrogen bonds between each molecule and each membrane model: (**a**) the upper layer of the myocardial membrane, (**b**) the lower layer of the myocardial membrane, (**c**) the upper layer of the ideal DSPC membrane, (**d**) the lower layer of the ideal DSPC membrane. The bars representing the hydrogen bonds of some salts with the myocardial membrane are annotated with the type of phospholipid that contributes the most to forming hydrogen bonds.

Hydrogen bonds form and break throughout the MD simulations. Anthracycline molecules can form hydrogen bonds within themselves, with neighboring water molecules, and with the myocardial membrane. Bonds that form between the molecules and the membrane head groups facilitate molecule anchorage to the membrane’s surface, seeding the cardiotoxic effect. Figure 3 illustrates the average number of hydrogen bonds created per each anthracycline molecule with each membrane model. We employ a widely used geometrical criterion to determine this number^26^. Specifically, a hydrogen bond exists when a donor (Do), connected to a polar hydrogen, and an acceptor (A), oxygen or nitrogen, are at a distance r_DoA_ ≤ 0.35 nm. Simultaneously, the hydrogen-donor–acceptor angle must be 30° or less.

Again, we find that the ideal DSPC membrane does not accurately reflect the distinctive behavior of the different molecules. In fact, for this ideal membrane, the average number of hydrogen bonds is very low (below 0.12) for all molecules. This is not the case for our myocardial membrane. Figure 3 confirms that a myocardial membrane's structural complexity and realistic composition are indeed required to represent the specificities of the different anthracycline behaviors. Moreover, in agreement with our residence time analysis, hydrogen bonding of the salts is significantly higher than for the metabolites, which is, in turn, typically higher than for the pristine forms. We interpret this direct correlation as indicating that the molecules are anchored with the help of hydrogen bonds, allowing them to spend more time near phospholipids that promote such bonding.

Marked on Fig. 3, DSPC is the main phospholipid contributor to hydrogen bonding in the myocardial membrane. Meanwhile, DOXH^+^ is the only molecule that forms hydrogen bonds equally with all phospholipids on the lower layer of the myocardial membrane. Those are DSPC, DSPE, and the acidic, negatively charged DSPS. Note that DSPC alone in the ideal membrane model could not form nearly as many hydrogen bonds with the cation molecules as it could in the realistic myocardial membrane, where the cooperative effect of neighboring phospholipids promotes such bonding. This finding further confirms our conclusions from the residence time analysis.

Another interesting observation is that the cationic molecules do not necessarily reside closer to the negatively charged DSPS in the lower layer of the realistic myocardial membrane to form hydrogen bonds. Instead, the cationic molecules prefer the zwitterion DSPC, when assisted by different types of neighboring phospholipids. This implies two important ideas. The first is that the cation anthracyclines cannot displace the Na^+^ ions that neutralize the DSPS phospholipids in the myocardial membrane^27,28^. Instead, they associate more with the zwitterion DSPC, which likely relates to the mechanism by which the different charged molecules bind to the phospholipids^28^. The second is that in a more complex and realistic membrane system, deductions based on simple electrostatic arguments are insufficient to characterize the interactions between the anthracyclines and the myocardial membrane. Instead, cooperative and dynamic interactions captured by MD simulations can accurately depict the more complex picture of the membrane/drug behavior. More details on the hydrogen bonds of the different drug molecules with each phospholipid are presented in Supplementary Table S3.

Additionally, details on the hydrogen bonds of the different drug molecules with water are presented in Supplementary Table S4. Of special interest is the enhancement of an anthracycline molecule's solubility when metabolized. During metabolism, the carbonyl group on carbon number 13 is hydroxylated into the more hydrophilic hydroxyl group^3^. When compared to their pristine counterparts, the metabolite structures do not only form more hydrogen bonds with the myocardial membrane, they also form more hydrogen bonds with the surrounding water molecules^4^. This finding implies that metabolite readiness to form hydrogen bonds with the myocardial membrane did not reduce their hydrogen interaction with water. In fact, their interaction with water was still higher than for the pristine forms, implying that metabolite molecules are generally more soluble in water, while also more interactive with the myocardial membrane^4^.

#### Diffusion coefficients and surface immobilization

**Figure 4:**
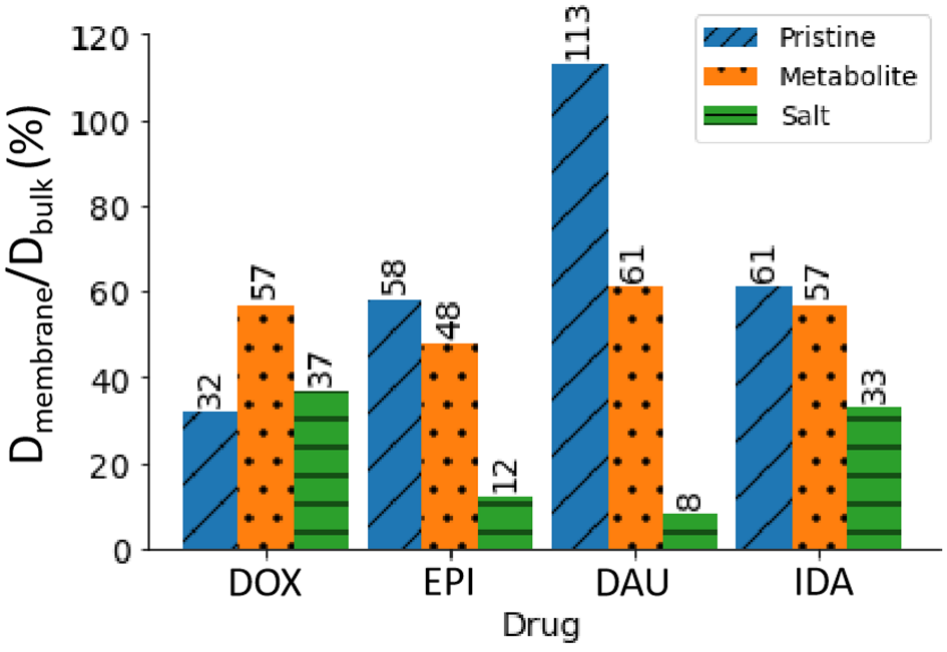
The percentage ratio between the drug diffusivity near the myocardial membrane D_membrane_ and its diffusivity in bulk water D_bulk_. The lowest *R*^2^ value of the linear fit of the mean square displacement data used to calculate D is 0.98.

To assess the ability of the myocardial membrane to immobilize the incoming anthracycline molecules, the diffusion coefficient of each molecule is calculated in bulk water and again in the proximity of the myocardial membrane. The percentage ratio between the diffusivities of the drugs near the myocardial membrane and their diffusivities in bulk water are shown in Fig. 4. The actual values along with the ones near the ideal DSPC membrane are available in Supplementary Fig. S4. These diffusivities were obtained according to Einstein equation from the slope of the mean square displacement of the molecules. More details are available in Supplementary Section S2. The experimentally determined diffusion coefficients in bulk-like water environment of DOX^29^, EPI^30^, and IDA^31^ are consistent with our calculated diffusivities of the same drugs in bulk water, and the comparison is shown in Supplementary Table S5. The ranking of the molecules from fastest to slowest is the same from both experiments and our computations (DOX > IDA > EPI)^29–31^. With the exception of pristine DAU, a significant decrease in the diffusion of all molecules is noted when molecules are moved from bulk water to the myocardial membrane environment which clearly indicates a membrane surface immobilization of these molecules. The exception observed in the case of DAU is probably within the uncertainty limitations of molecular simulations and we do not recommend a conclusion that DAU speeds up near the myocardial membrane. This is since the difference in its bulk water diffusivity and near-membrane diffusivity is small. However, further attention should be directed towards DAU’s diffusive ability both experimentally and computationally.

This remarkable slowing down of the molecules increases the chances of interaction between the molecules and the myocardial bilayer, which in turn contributes to “potential” cardiotoxicity. EPIH^+^ and DAUH^+^ are the two molecules that slow down the most in the presence of the myocardial membrane, falling in magnitude 8- and 12-folds, respectively. These two molecules are trapped within the upper layer of the membrane as depicted earlier by the residence time and hydrogen bond analyses. DOX and IDAH^+^ slow down to less than 1/3 of their diffusion coefficient in bulk water. A more detailed assessment of the molecules’ diffusion in all dimensions is represented in Supplementary Table S6, where we can see that the diffusion coefficient in the z-direction perpendicular to the plane of the membrane is slowed down significantly in clear indication of the surface immobilization effect.

#### The average location of the molecules from the mid-plane of the bilayer

An assessment of the ability of anthracyclines to cause cardiotoxicity is the molecules’ permeation into the myocardial bilayer (or through it). For our unbiased MD simulations, the molecules' complete crossing of the bilayer cannot be observed. As such, we compute the molecules' average position relative to the bilayer’s (myocardial membrane’s) mid-plane, which separates both layers and is parallel to them. We note that this computation is done twice for each molecule; once when it is present underneath the lower layer and another time when it is above the upper layer. We remark that the mere relocation of a drug molecule from above the upper layer to below the lower one does not imply membrane crossing. Rather, it follows from the periodic boundary conditions imposed on the simulation cell. The closer a molecule is to the mid-plane, the likelier its permeation into the member and the higher its cardiotoxic probability. Details on the relevant calculations are provided in Supplementary Section S2.

**Figure 5:**
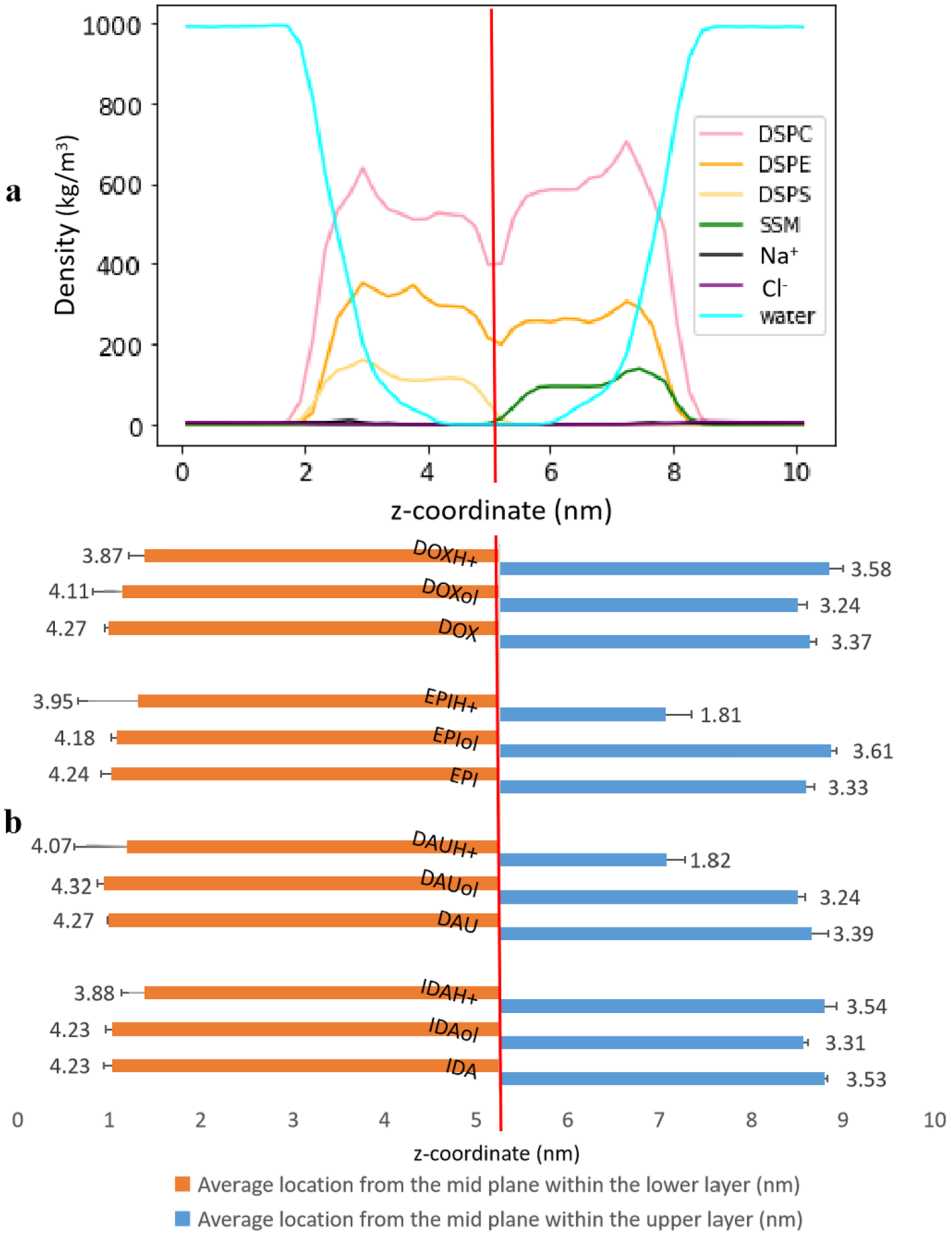
(**a**) The one dimensional density profile of the myocardial membrane in (kg/m^3^) and (**b**) a graphical representation of the average location in (nm) of each anthracycline molecule from the mid plane of the bilayer membrane at the upper layer (blue bars) and at the lower layer (orange bars). The edge of the bars show the location of the molecules with respect to the mid plane of the bilayer myocardial membrane. The numerical annotation is the distance in (nm) between the molecules’ average locations and the mid plane. The vertical red line in panels (**a**) and (**b**) represents the mid plane. The error bars represent the standard deviation which is calculated by dividing last 800 ns of the trajectory into 4 blocks.

Figure 5a illustrates a one-dimensional density profile of the myocardial membrane constituents as a function of the *z*-coordinate (nm), measured from the mid-plane. The average z-coordinate of each molecule within the upper and lower layers is shown in Fig. 5b. A similar representation of molecule positions in the idealized DSPC membrane is shown in Supplementary Fig. S5. Generally, all the molecules exhibit greater permeation affinity toward the upper layer than the lower layer in the myocardial membrane. This could be owing to the upper layer’s lipid tails rather than head groups. Specifically, the lipid tails associated with the SSM head groups in the upper layer contain one carbon double bond, which is not the case for the lower layer. This unsaturation makes the upper layer in our myocardial membrane model more fluid than the lower layer. However, this fluidity alone is insufficient to conclude easier crossing of the anthracyclines through the upper layer. One needs to consider other factors, such as hydrogen bonding, residence time, and surface immobilization, in combination with proximity to the mid-plane of the bilayer structure to obtain a full picture of the drug’s ability to cross or to be trapped in the membrane.

This analysis tool further lends insight into the behavior of the salt molecules, especially EPIH^+^ and DAUH^+^. They were both able to diffuse into the membrane by interacting with the upper layer. They were also able to reach within 1.8 nm of the mid-plane of the myocardial membrane. This finding implies that, within the unbiased MD simulation time, these two cations succeeded in overcoming the first energy barrier presented by the head groups of the phospholipids of the upper layer. However, the second energy barrier of the lipid tails was too high for the molecules to cross during the simulation time. This is depicted in Supplementary Fig. S6, which illustrates the free energy profiles of EPIH^+^ and DAUH^+^ across the membrane. These two different barriers are in alignment with the conclusions reported by Yacoub et al. on DOX crossing^6^.

## Discussion

The results presented in the previous section are combined here to understand how all the factors taken together rank the drugs in terms of their potential to induce cardiotoxicity. We hypothesize that a molecule first anchors to the membrane to prepare itself for either crossing or storage within the membrane, which eventually leads to cardiotoxic action. We rank the molecules according to their ability to anchor at and partially permeate the membrane. For this ranking, we developed a set of ad hoc criteria based on the four factors analyzed in the “Results” section. Our criteria and rankings are presented in Table 2. As seen, the cation forms of the salts have the highest potential for cardiotoxicity. The metabolites follow, ranking second. Finally, out of the pristine forms, DOX is potentially the most toxic molecule, ranking in our study even higher than the metabolite IDAol.

Toroz and Gould reported the insertion speed of the 4 pristine anthracycline molecules across their model membrane as follows: EPI < IDA < DAU < DOX^20^. Our analysis of the pristine forms agrees in the fact that DOX has the highest potential of inducing cardiotoxicity since its diffusivity is reduced near the myocardial membrane. Meanwhile, our analysis cannot distinguish among the pristine EPI, IDA, DAU.

Next, we compare our ranking from Table 2 to clinical data from literature. Unfortunately, only the metabolite molecular form of the drugs is clearly distinguished in literature from its pristine and protonated counterparts. In fact, the term “Doxorubicin” for example is used interchangeably to describe both the pristine and salt forms of the drug in past studies. In this study, however, we emphasize the distinction between all the different molecular forms that exist physiologically. Beginning with the clearly distinguished metabolite forms, they are widely reported as being the more cardiotoxic molecular form of the drugs^3,4,32^. According to our results, this is true when the potential of cardiotoxicity of the metabolites is compared to that of the pristine forms. However, when compared to the salt forms, the metabolites have lower chances of damaging the heart cells.

**Table 2:** Summary of the ranking of the anthracyclines according to their potential to induce cardiotoxicity. For the molecule to be highly cardiotoxic, it has to appear as a first category in as many criteria as possible. The criteria are (1) According to residence time; if the molecule resides more than 20% of the simulation time near the myocardial membrane it is considered 1st category, and if between 12 and 20% it is considered 2nd category. (2) According to the average number of hydrogen bonds with the myocardial membrane; if more than 0.1, then it is 1st category and if between 0.05 and 0.1, then it is a 2nd category molecule. (3) According to the distance from the mid plane parallel to the myocardial membrane; if less than 2.6 nm, it is considered a 1st category molecule and if between 2.6 nm and 4.16 nm, it is considered a 2nd category. (4) According to the reduction in the diffusion coefficient of the molecule near the myocardial membrane in comparison to the diffusivity in bulk water; 1st category for molecules whose diffusivity drops to less than 25% of their bulk values and 2nd category for molecules whose diffusivity drops to the range from 25 to 35% of their values in bulk water. In the table; 1st category is represented by the symbol *, 2nd category is represented by the symbol × . Empty box is used when the drug does not fall in any of these two categories. “Both” indicates that the criteria is observed in both layers of the membrane, “L” indicates that the criteria is observed in the lower layer only and “U in the upper layer only.

	Residence time	Average number of hydrogen bonds	Distance from mid plane parallel to the membrane	Reduction in diffusion coefficient
1st category	*	*	*	*
2nd category	×	×	×	×
Neither 1st nor 2nd category				
Drugs’ ranking				
EPIH^+^	B*	U*	U*	*
DAUH^+^	U*	U*	U*	*
IDAH^+^	L*	L*		×
DOXH^+^	L*	L*		
DOXol	B ×	L ×		
EPIol	B ×	L ×		
DAUol	U ×			
DOX				×
IDAol, EPI, DAU, IDA				

There is not enough data in the literature that shows a clear comparison between the concentrations of the different molecular forms of anthracyclines that exist inside the body as a function of time. Future experimental work is encouraged to direct attention to the salt, pristine, and all metabolite forms of anthracyclines that are physiologically present over the course of the years during and after treatment. Moreover, future computational studies are prompted to use more complex myocardial membrane models that depict experimental findings as closely as possible. This includes incorporating cholesterol, calcium channels, and membrane proteins.

It is desirable to have a simple metric to rank the cardiotoxicity of drugs. This metric has to be efficiently computed and used in screening studies. The dipole moment of the molecules was proposed as such a metric^20^. We used both CHARMM force field and density functional theory (DFT) calculations to compute the dipole moment for each molecule, as provided in Supplementary Fig. S6. Unfortunately, we found no correlation between the dipole moment and the ranking presented here. This indicates that until a simple metric is discovered, the hard work of simulating the drug/cell interaction is needed to decipher the details of the cardiotoxic action.

## Conclusions

We employ unbiased classical molecular dynamics simulations to assess the interactions that take place between 12 anthracycline molecular forms and the myocardial membrane. We focus on 4 drugs: Doxorubicin, Epirubicin, Daunrubicin, and Idarubicin in three physiological molecular forms: pristine, major metabolite, and cation. We assess the interactions in terms of (i) residence time near the myocardial membrane, (ii) average number of hydrogen bonds formed, (iii) surface immobilization and diffusion coefficient, and (iv) drug molecule distance from the mid-plane of the membrane. Combining these parameters, we compare the ability of our proposed myocardial membrane model, featuring more realistic, experimentally based structures, to a simpler model prevalent in the literature, in capturing the behavior of the molecules. Our results demonstrate that our realistic membrane model captures behaviors that cannot be observed using the ideal model made out of one phospholipid type. Specifically, our model highlights that the different anthracycline drugs can be more attracted toward different membrane layers when in salt form. At the same time, the drugs permeation of the upper layer in every form is typically easier than the lower layer. These findings confirm that cardiotoxic mechanisms are membrane-layer dependent and drug-form dependent, rendering their study rather complex and needing further in-silico and experimental investigations. Moreover, future computational work can expand on the 4 metrics presented here to understand the drug/membrane interaction by including further metrics such as the changes in the surface tension of the membrane layers upon drug insertion, the activation barriers for drug crossing the layers of the membranes, and the lifetimes of hydrogen bonds that form between the drug and the membrane.

The ranking of the anthracycline forms from the most to the least potentially cardiotoxic molecular places salt forms in the lead, followed by secondary alcohol metabolites, then pristine forms. Interestingly, the pristine form of Doxorubicin ranked as potentially more cardiotoxic even higher than all other pristine forms and even higher than the metabolite form Idarubicinol, which again warrants further experimental research for confirmation. Finally, we confirmed that the dipole moment of the molecule cannot serve as a simple metric for its potential cardiotoxicity. Our work urges future research to include more realistic features of the cell membrane, recognize the importance of differentiating among the same forms of the same drug, and to seek an optimum balance between the efficacy of treating cancer and the mitigation of cardiotoxicity.

### Methods

Details about the preparation of the membrane and anthracycline molecules are presented here. These include the initial structures of the molecules and the phospholipids, the tools and software used, molecular dynamics parameters and protocol, and density functional theory details.

### Membrane and drug molecules structure preparation

We believe that previously reported approaches overly simplify the inherent complexity of the myocardium where several phospholipids are present in unique arrangements^22^. Our attempt at creating a more reliable membrane model included the construction of several test systems. Those were assessed in terms of symmetry among the lower and upper membrane layers, temperature effect, size of the membrane, hydration ratio, and resemblance to the heart cell membrane as determined experimentally^22^.

All membrane models were constructed using the Membrane Builder tool on CHARMM-GUI web server^33,34^. The final realistic and representative membrane system that was used in this study was an asymmetrical bilayer of 128 phospholipids of 4 different head groups, shown in Fig. 1. The ratio of each phospholipid was selected according to the experimental findings of Matos et al.^22^ and are shown in Table 1. As seen in the table, SSM is only present in the upper layer of the membrane. It is also the only phospholipid head group that contains a double bond in one of its lipid tails. The double bond lies between carbons number 4 and 5 and both lipid chains are 18 carbons-long. The lipid tails for all other head groups were kept the same: saturated chains of 18 carbons. This difference in the saturation of the lipid tails is simply due to the nature of sphingomyelin carbon chains where either one chain is longer than the other or an unsaturation is present. We chose the latter option to keep at least the lengths of all lipid chains constant in our system. Moreover, DSPS is exclusive to the lower layer. It is also the only negatively charged head group. The ratio of water to phospholipid molecules is around 50:1. Salt concentration is 0.15 mol/L NaCl and temperature is 310 K. This model was chosen because it displayed the best fluidity and stability among all candidates as discussed in the “Results” section.

The structure of each drug molecule was obtained from Research Collaboratory for Structural Bioinformatics (RSCB) Protein Data Bank (PDB)^35^. The chemical ID’s were DM11, DM21, DM51, and DM61 for Daunorubicin, Doxorubicin, Idarubicin, and Epirubicin, respectively. Using Avogadro software^36^, the files were manipulated to create three different forms of each molecule: pristine, protonated cation (salt form) and major metabolite. The pristine forms were obtained using a pH of 7.5 on Avogadro while salt forms were produced when the pH was lowered to 5. Primary metabolites were created by adding a hydrogen atom to the oxygen atom of the carbonyl group on carbon number 13 of the anthracyclines, making it a hydroxyl group.

### Molecular dynamics (MD) simulations protocol

The drug molecules interactions were described using CHARMM-General Force Field, CGenFF version 2.4.0.^37,38^. As for the phospholipids, CHARMM36 is the force field of choice in this study^39^ GROMACS software package version 2020.6 was used to run all MD simulations^40,41^.

The first set of simulations were the drug molecules solvated in bulk water. Each molecule was separately added to a 5.5 nm × 5.5 nm × 5.5 nm cubic box and solvated in liquid water. It was then energy-minimized using the steepest descent algorithm and the Verlet scheme was used to update the neighbor list. Next, two consecutive equilibrations were performed using NVT ( N = constant number of particles, V = constant volume, and T = constant temperature) ensemble for 1 ns then an NPT (P = constant pressure) ensemble for 5 ns. During equilibration, the hydrogen bonds were constrained using LINCS algorithm. Finally, each molecule was simulated in an NPT ensemble for 100 ns. The time step used is 2 fs and the writing frequency is one configuration every 1 ps. A Nosé-Hoover thermostat is used in the NVT ensemble, while during the NPT simulations we employed a Parrinello-Rahman barostat. The temperature was set to 310 K, and the pressure was set to 1 atm and periodic boundary conditions were set in all three dimensions. Particle-Mesh-Ewald (PME) is used for coulomb interaction calculations beyond the cutoff value of 1.2 nm. The CHARMM-modified water model TIP3P is used to represent water molecules. Like the drug molecules, the myocardial membrane was separately simulated in bulk water. It was minimized for 5000 steps using steepest descent with LINCS constraints. This was followed by CHARMM-GUI’s standard 6-step consecutive equilibrations. The first two equilibrations followed an NVT ensemble for 0.125 ns each. For equilibrations 3 to 6, an NPT ensemble is maintained using a Brendsen thermostat and barostat. Equilibrations 3, 4, 5 & 6 were simulated for 0.125 ns, 0.5 ns, 0.5 ns, and 20 ns respectively. Each equilibration step applied less constraints on the lipids until no constraints were involved in the last one. The membrane’s production MD run was simulated for 200 ns. The temperature was controlled by a Nosé-Hoover thermostat while pressure was maintained at 1 atm using a Parrinello-Rahman barostat. All other parameters were the same as those used for the drug molecules in bulk water. Finally, 13 more simulations were performed. The first one was an extended membrane-only simulation that ran for 1.2 μs. The other 12 simulations were of the 12 drug molecules separately added to the membrane. The drugs were initially positioned around 13 Å away from the upper layer of the membrane. Each simulation ran for 1.2 μs. It is worth noting that the ideal DSPC membrane and its interaction with the 12 molecules were simulated in exactly the same protocol as the myocardial membrane.

### Density functional theory (DFT) protocol

CRYSTAL17 software package was utilized for the DFT calculations of the drug molecules^42^. Exchange–correlation interactions were represented using the M06-2X functional. Electronic wave functions were expanded using a double zeta correlation-consistent polarized valence basis set (cc-PVDZ). The molecules were fully optimized with no constraints using an energy convergence criteria of ΔE ≤ 10^−8^ Hartree. The input coordinates for each molecule were obtained from an MD minimization in vacuum. The dipole moments of the optimized molecular geometries in vacuum are available in Supplementary Table S7. It is worth noting that dipole moments were calculated for the charge neutral species only, i.e. pristine and primary metabolite forms.

### Supplementary Information

Supplementary Information.

Supplementary Video 1.

Supplementary Video 2.

## Data Availability

The datasets generated during and/or analyzed during the current study are available from the corresponding author on reasonable request.
