# Supplementary material for: Molecular assessment of drug-phospholipid interactions consequent to cancer treatment: a study of anthracycline-induced cardiotoxicity
Source: Sci Rep. 2023 Dec 13;13:22155. doi: 10.1038/s41598-023-48184-4 (PMC10719326; doi:10.1038/s41598-023-48184-4)
Supplement: Supplementary file 1 — Supplementary Information. [file 41598_2023_48184_MOESM1_ESM.docx]

**Supplementary Information for**

Molecular Assessment of Drug-Phospholipid Interactions Consequent to Cancer Treatment: A Study of Anthracycline-Induced Cardiotoxicity

Yara Ahmed^1^, Khalil I. Elkhodary^2^, Mostafa Youssef^2^ *

*Corresponding Author: mostafa.youssef@aucegypt.edu

^1^ Nanotechnology Program, The American University in Cairo, AUC Avenue, P.O. Box 74, New Cairo 11835, Egypt.

^2^ Department of Mechanical Engineering, The American University in Cairo, AUC Avenue, P.O. Box 74, New Cairo 11835, Egypt.

**This PDF includes:**

Supplementary information text – Section S1 and S2
Figures S1 to S6
Tables S1 to S7
Movies S1 and S2
SI References

Supplementary Information Text

**Section S1. Monitoring Simulation Parameters**

Analysis on the stability of the simulated myocardial membrane systems is conducted in terms of thermodynamic factors and root mean square deviation (RMSD) of the molecules in the systems. Analyses for the ideal (DSPC) membrane systems are not shown here but they show similar behavior. The 12 anthracycline molecules are presented in

**Figure S1.** The 12 anthracycline molecules. First column shows the pristine forms, second column shows the metabolites, and the third column shows the salt cation forms. (a to c) Doxorubicin, (d to f) Epirubicin, (g to i) Daunorubicin, and (j to l) Idarubicin. below. The averages of temperature, pressure, volume, and total energy of all systems remained stable throughout the 1.2 μs simulation duration as shown in **Figure S2** below. RMSD of the myocardial membrane system was an average of approximately 2 nm in all simulations, indicating minor oscillations and overall integrity of the membrane. RMSD of the drug molecules, shown in **Figure S3** below, and RMSD of water molecules (not shown) were higher, capturing the free movement of the molecules. All systems were stable and reliable for further examinations. The size of the drug molecules in terms of the radius of gyration (R_g_) was examined before and after being added to the membrane system. No significant changes in molecules’ sizes were observed between a bulk water environment and the myocardial membrane environment. All molecules averaged around a radius of 0.48 nm as shown in **Table S*1*.** Radius of gyration (R_g_) in nm of anthracylcine molecules in bulk water and in proximity to the myocardial membrane.

**Section S2. Simulation Analyses**

**Diffusion Coefficient Calculations**

The gmx msd command on GROMACS was used to calculate the mean square displacement (MSD) of the molecules from their initial positions. The resulting output file was plotted as MSD in nm^2^ as a function of time in ns. For the simulations in bulk water whose duration was 100 ns, only the first 30 ns were used in evaluating the diffusion coefficient since they exhibited a linear behavior and their statistics were reliable. Meanwhile for the membrane systems simulations which lasted for 1.2 μs, the first 0.2 μs were considered equilibration and the next 30 ns (between 0.20 μs and 0.23 μs) were used to calculate the diffusion coefficient again since the behavior was linear and the statistics were reliable. The slope of the linear region was calculated, and according to Einstein equation, was used to get the value of the diffusion coefficient (D). The values of diffusion coefficient of all molecules is shown in **Error! Reference source not found.**.
To calculate the average D in all dimensions, D = $\frac{1}{6}$*slope of linear region of average MSD. To calculate D in one dimension (e.g. x or y), D = ½*slope of linear region in that dimension.

**The average location of the molecules from the mid plane of the bilayer myocardial membrane**

First, the one-dimensional density profiles of the components of the myocardial membrane were computed using the gmx density command. From these profiles, we pinpointed the coordinates of the mid plane that separates both layers of the membrane. Then, the average location of the molecule above the upper layer < *z_up_* > was calculated as follows:

$<z_{up}> = \frac{\int_{z_{mid}}^{z_{cell}} z\rho_{drug}\left( z \right)dz}{\int_{z_{mid}}^{z_{cell}} \rho_{drug}\left( z \right)dz}$ ,

where *z_cell_*  is the z coordinate of the upper boundary of the simulation cell, *z_mid_* is the z coordinate of the mid plane, and ρ_drug_(z) is the one dimensional mass density profile of the drug.

In a similar fashion, the average location of the molecule below the lower layer < *z_low_* > was calculated as follows:

$<z_{low}> = \frac{\int_{0}^{z_{mid}} z\rho_{drug}\left( z \right)dz}{\int_{0}^{z_{mid}} \rho_{drug}\left( z \right)dz}$ .


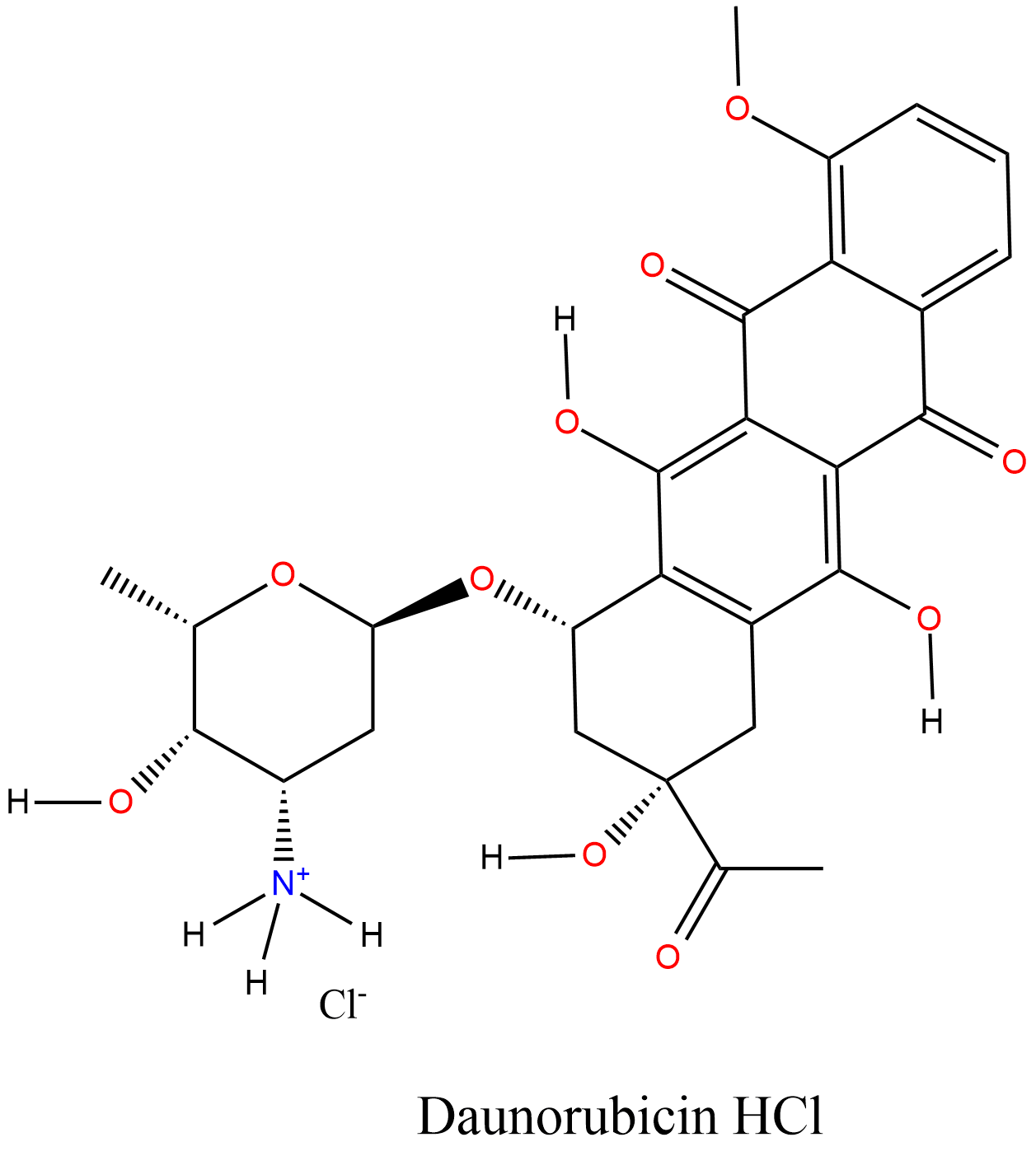

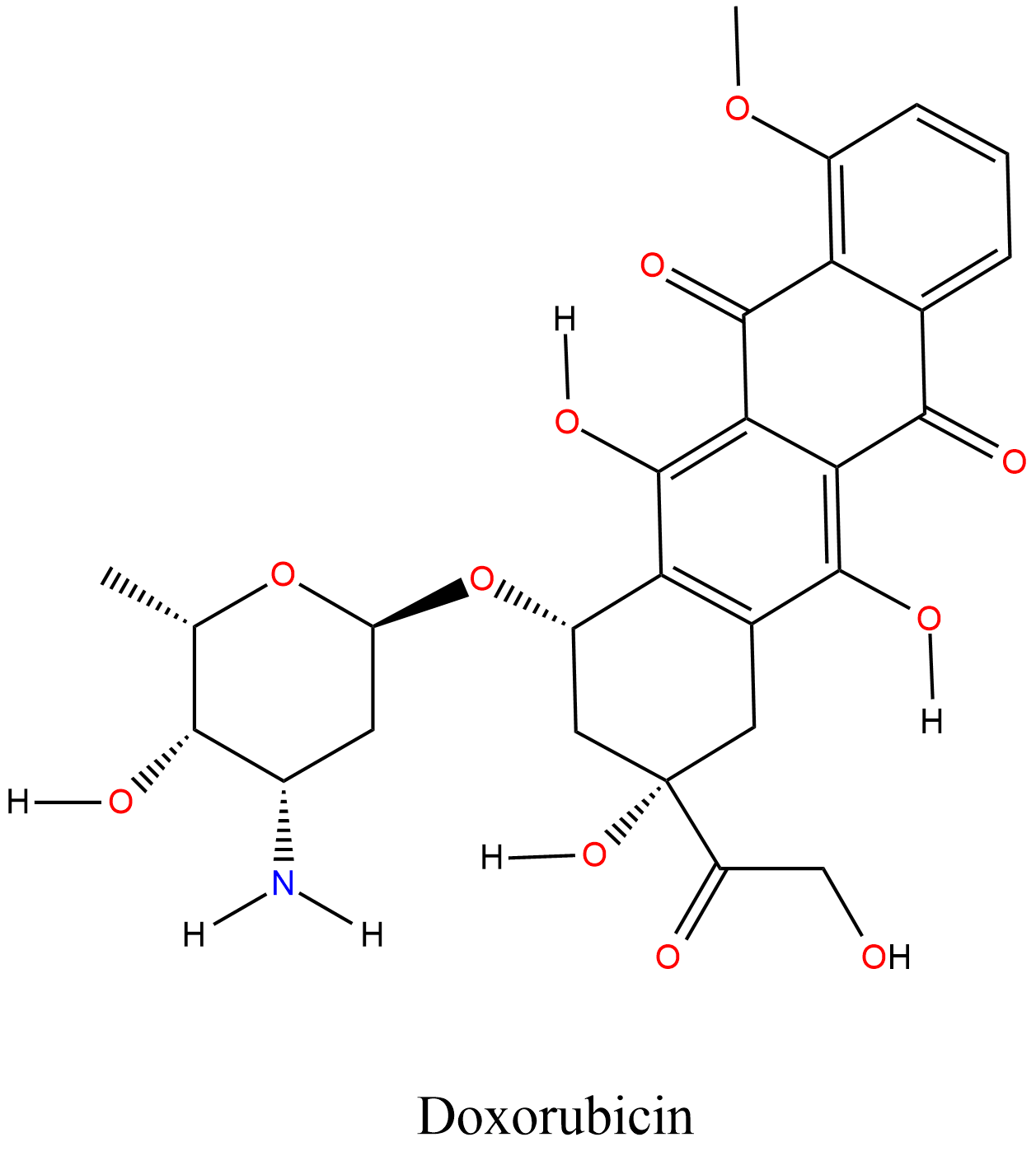

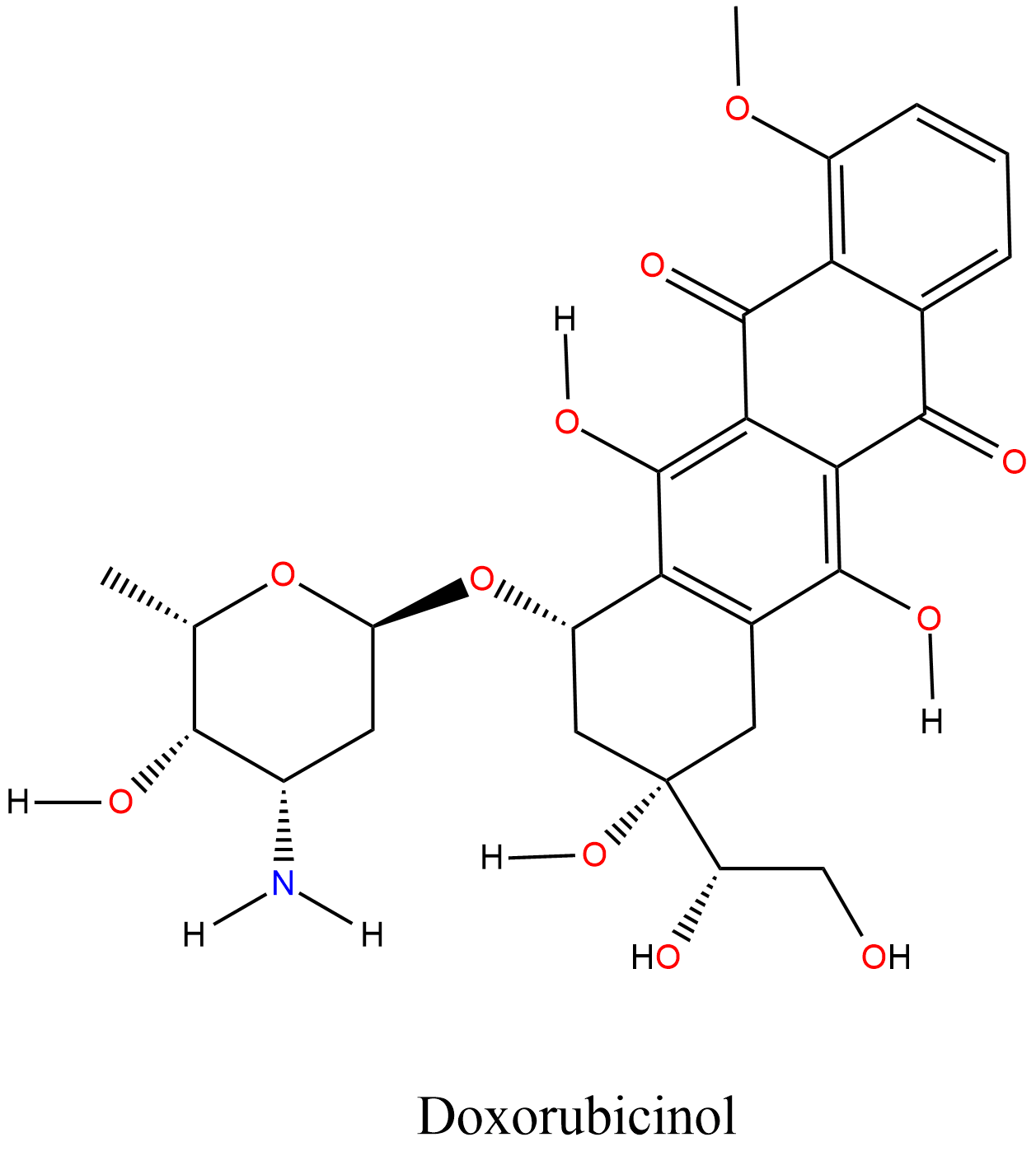


**a**

**b**

**c**


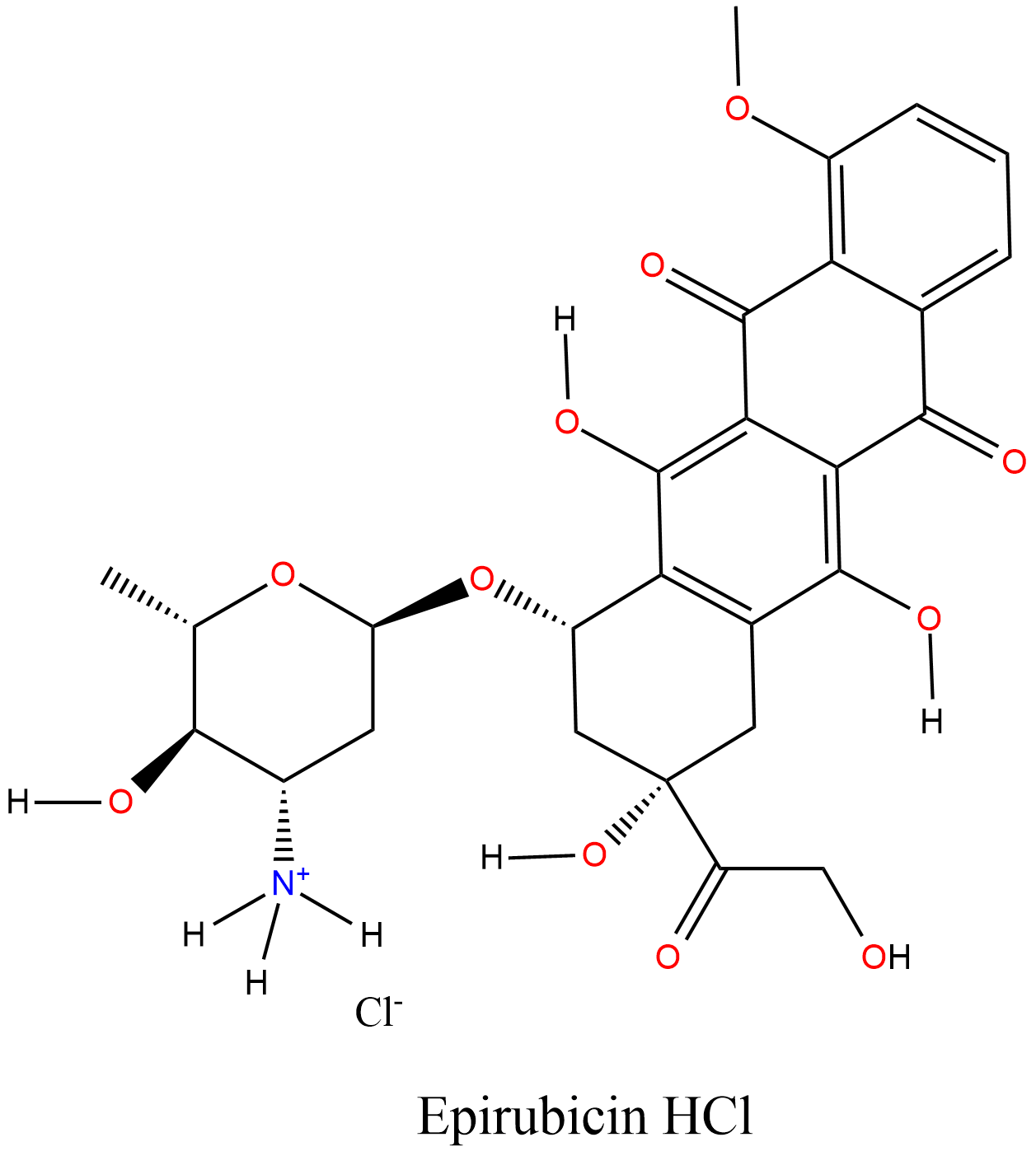

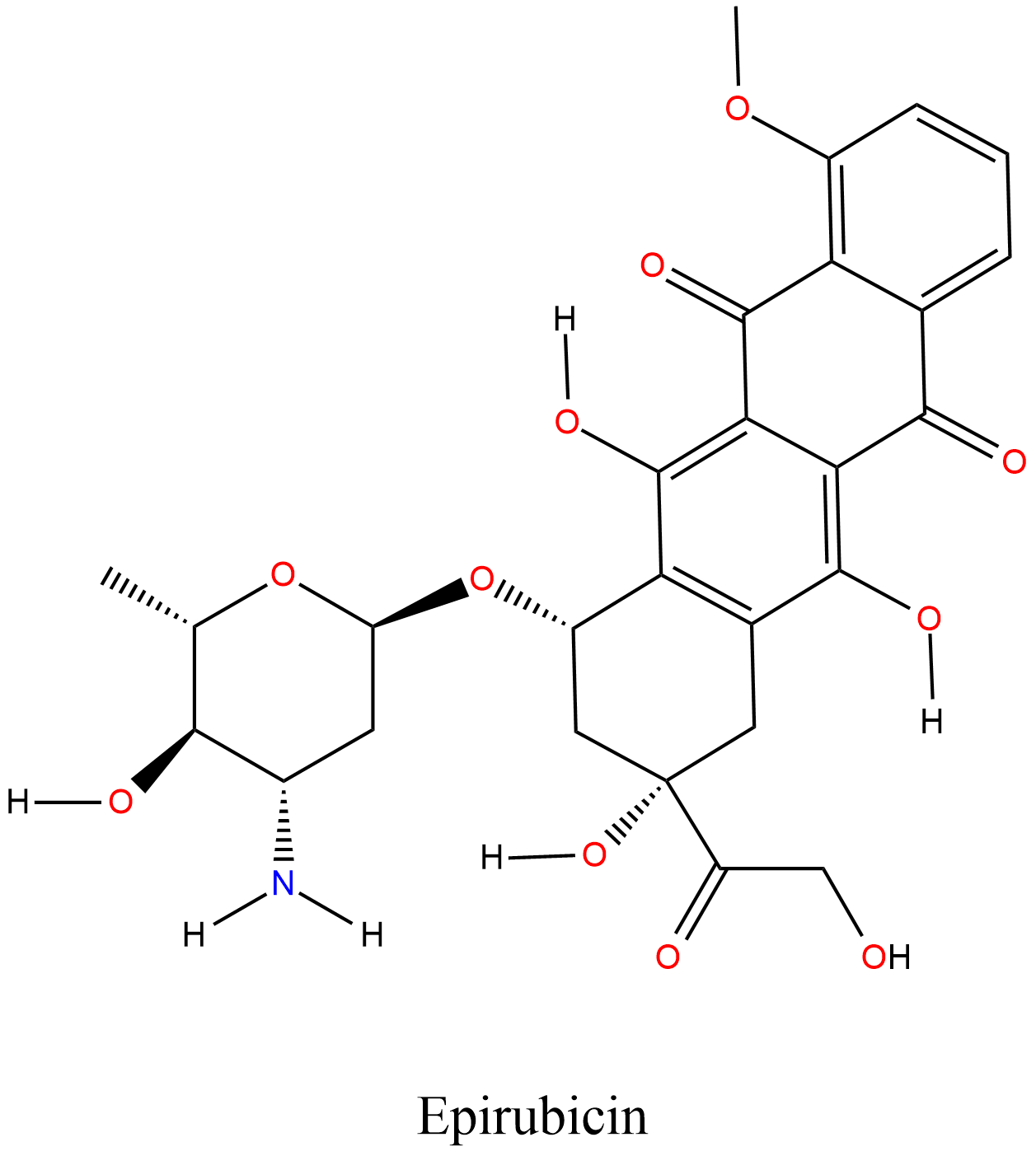

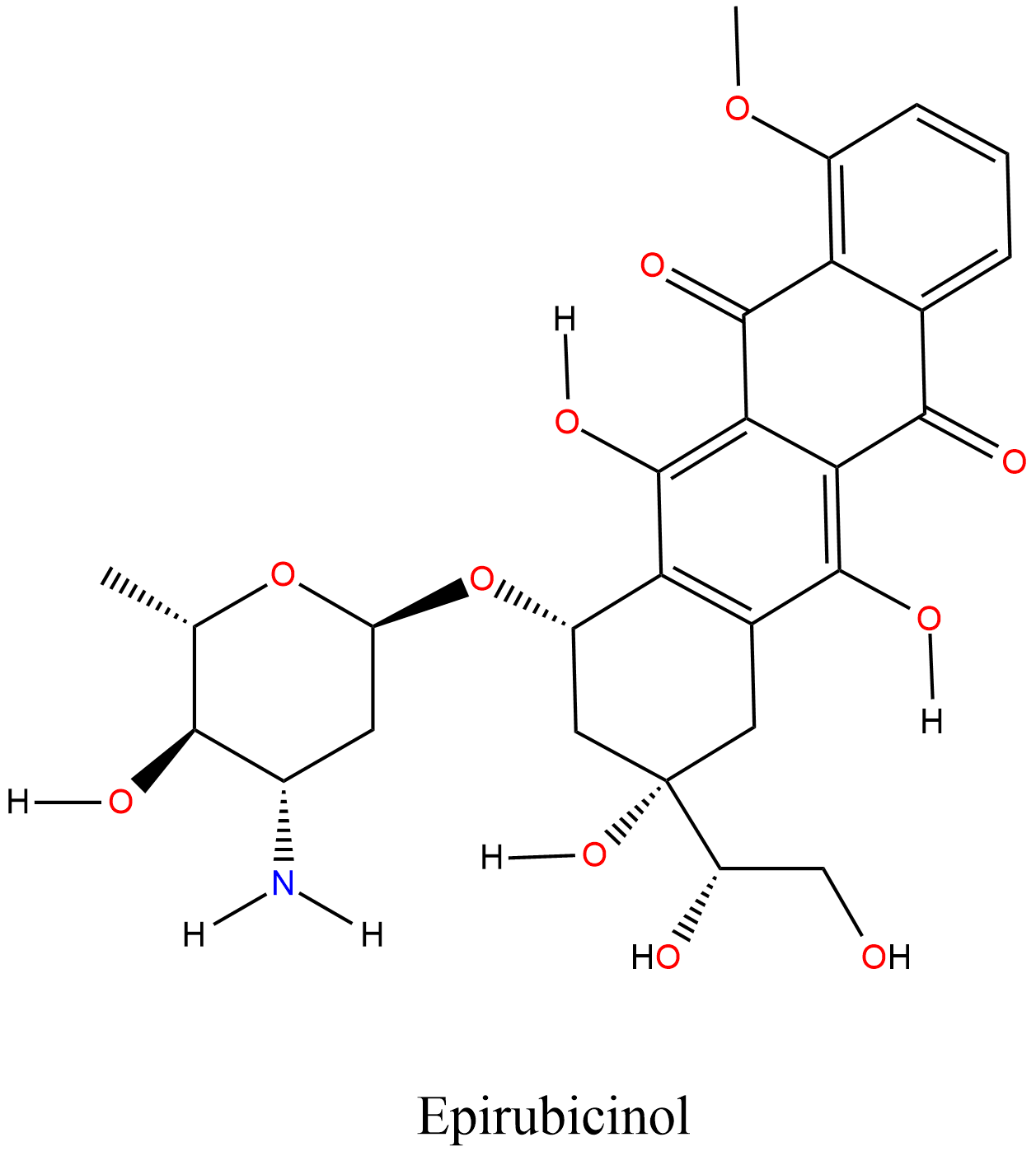


**d**

**e**

**f**


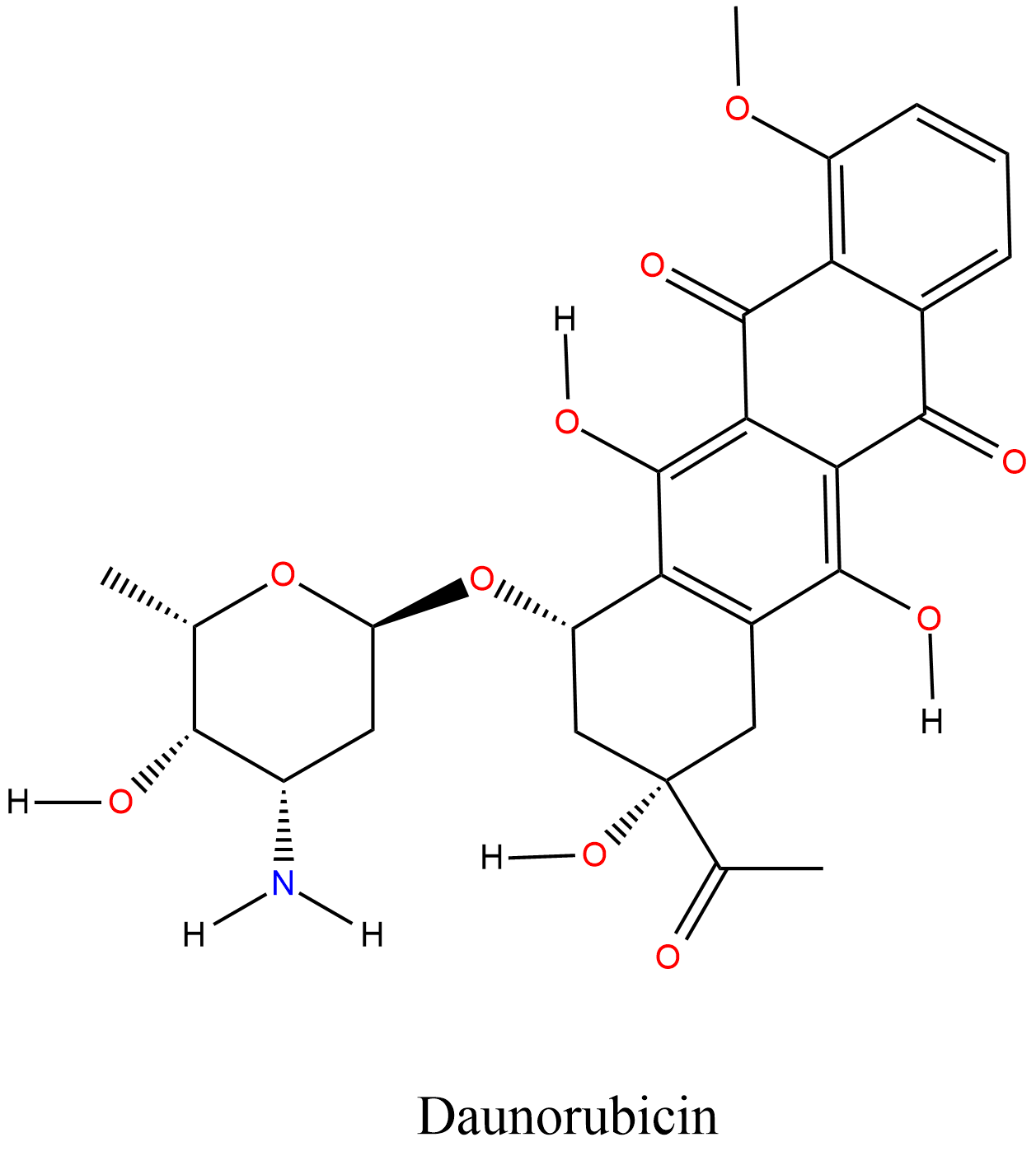

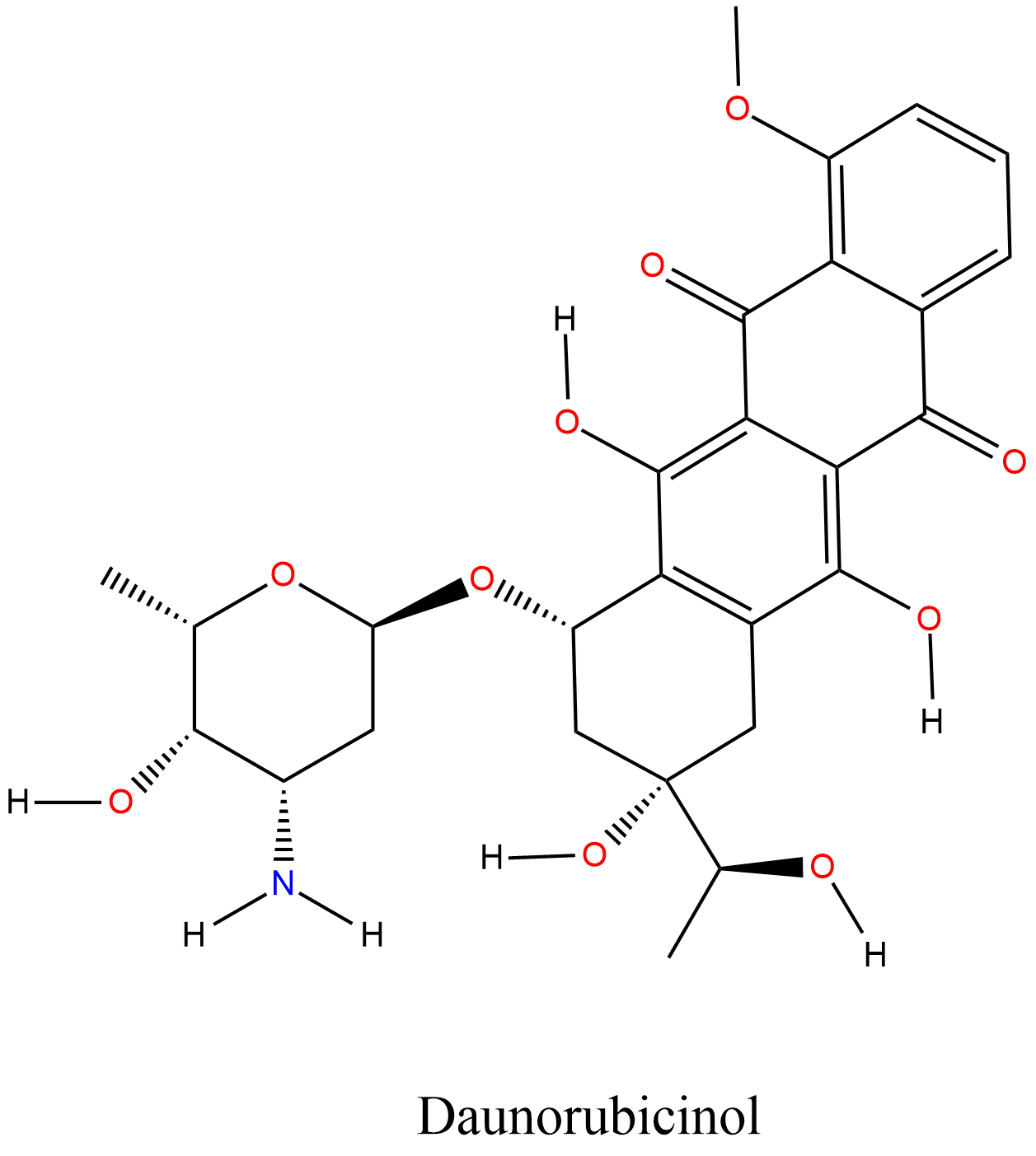

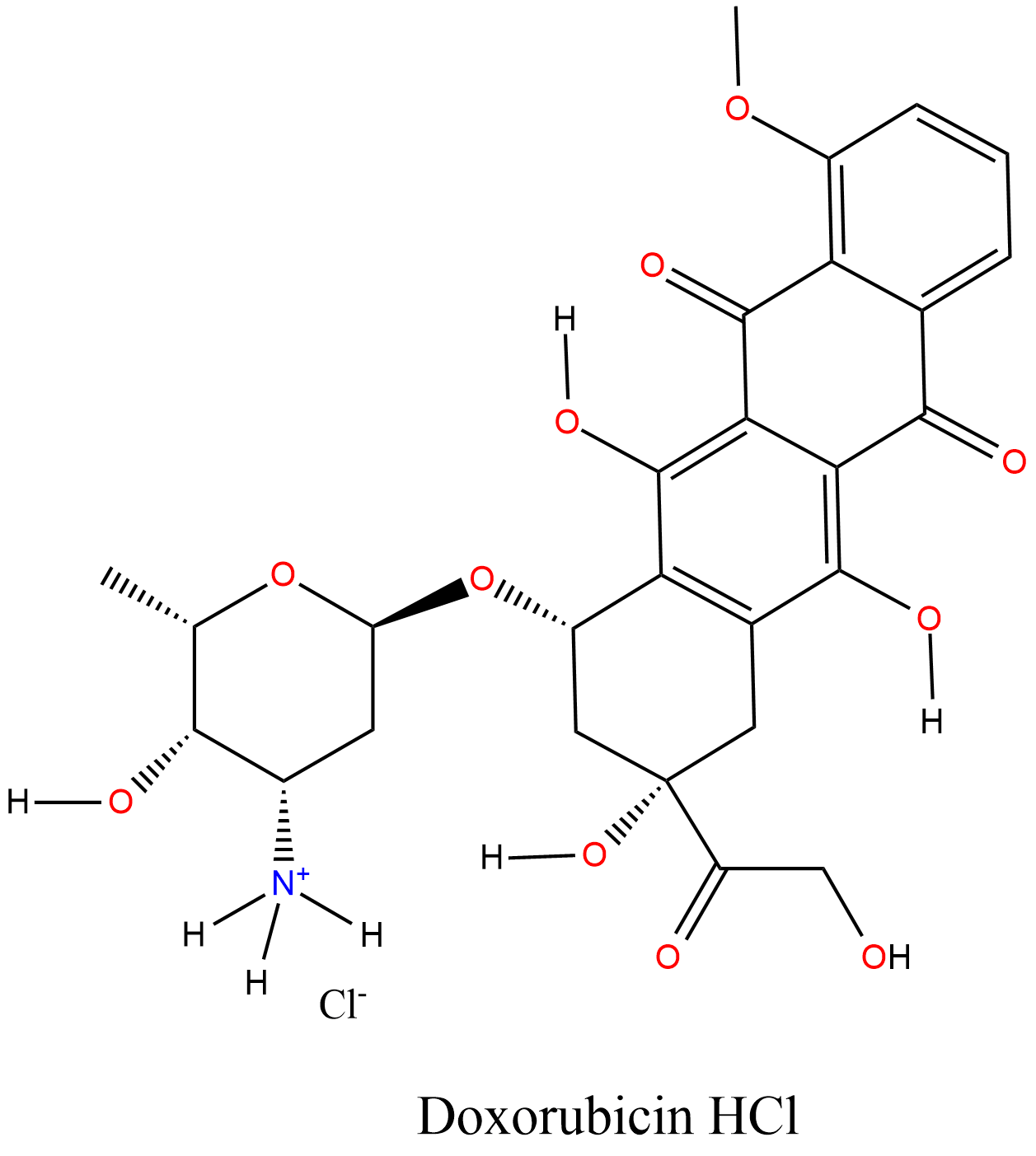


**h**

**i**


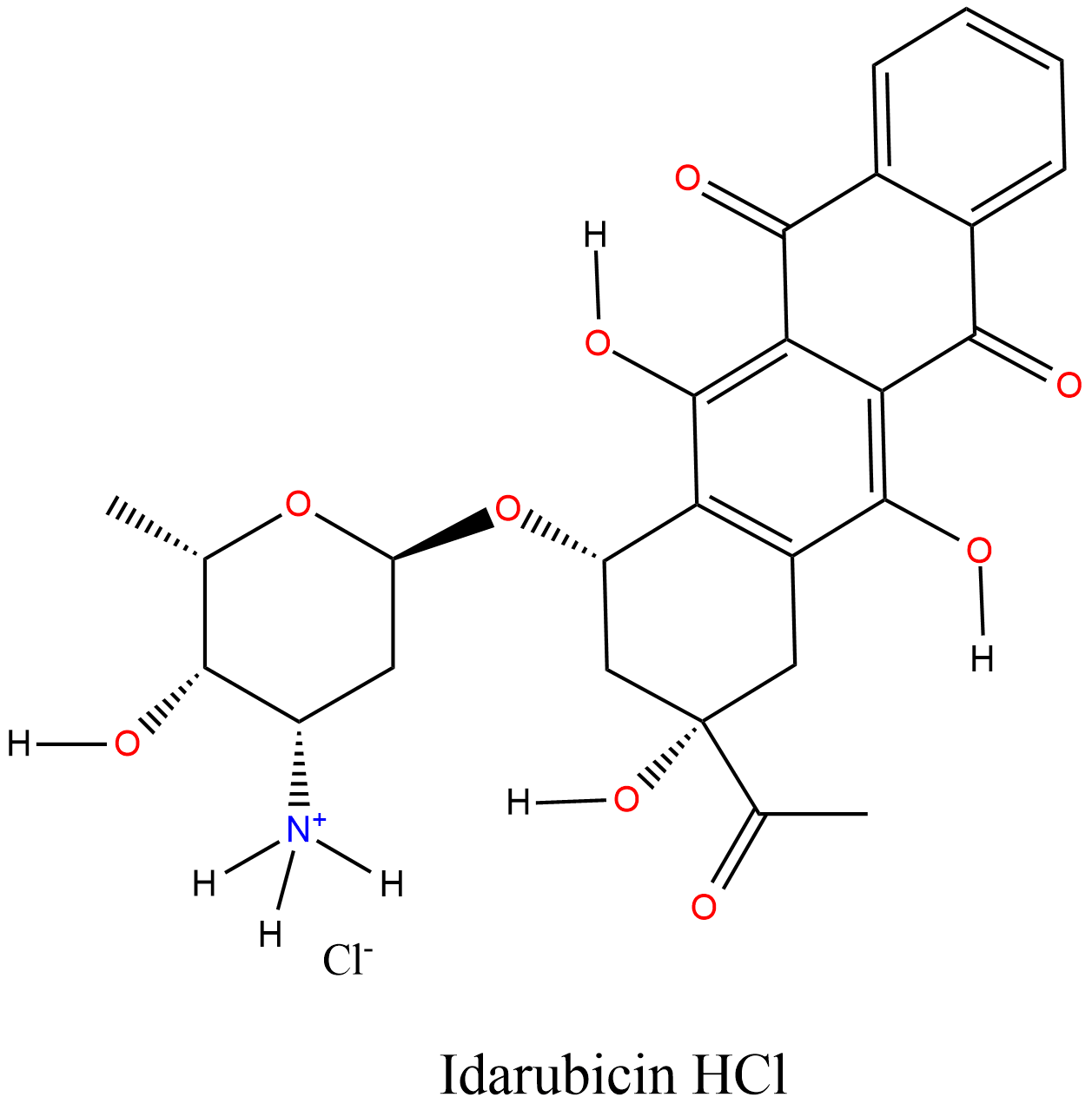

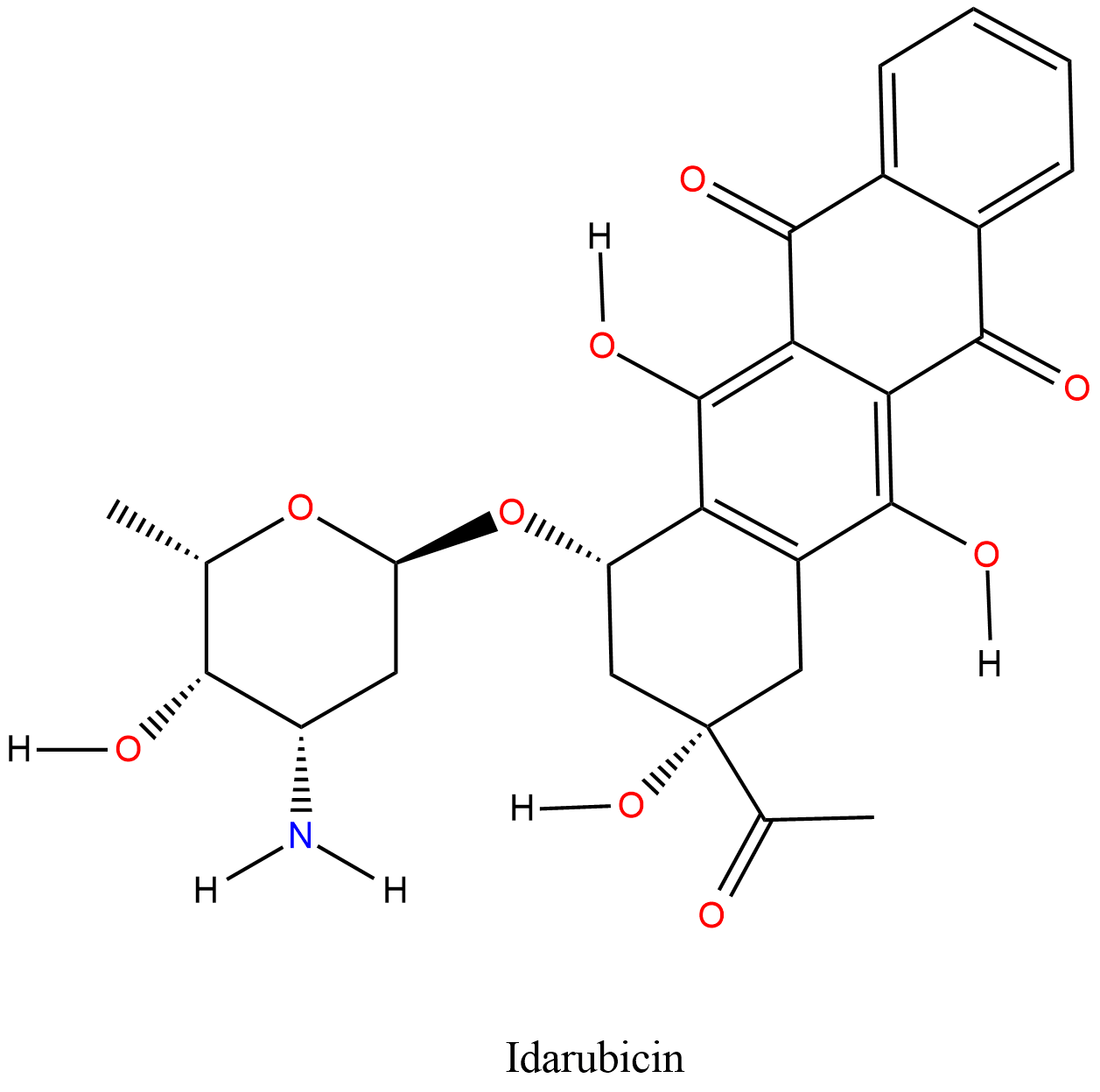

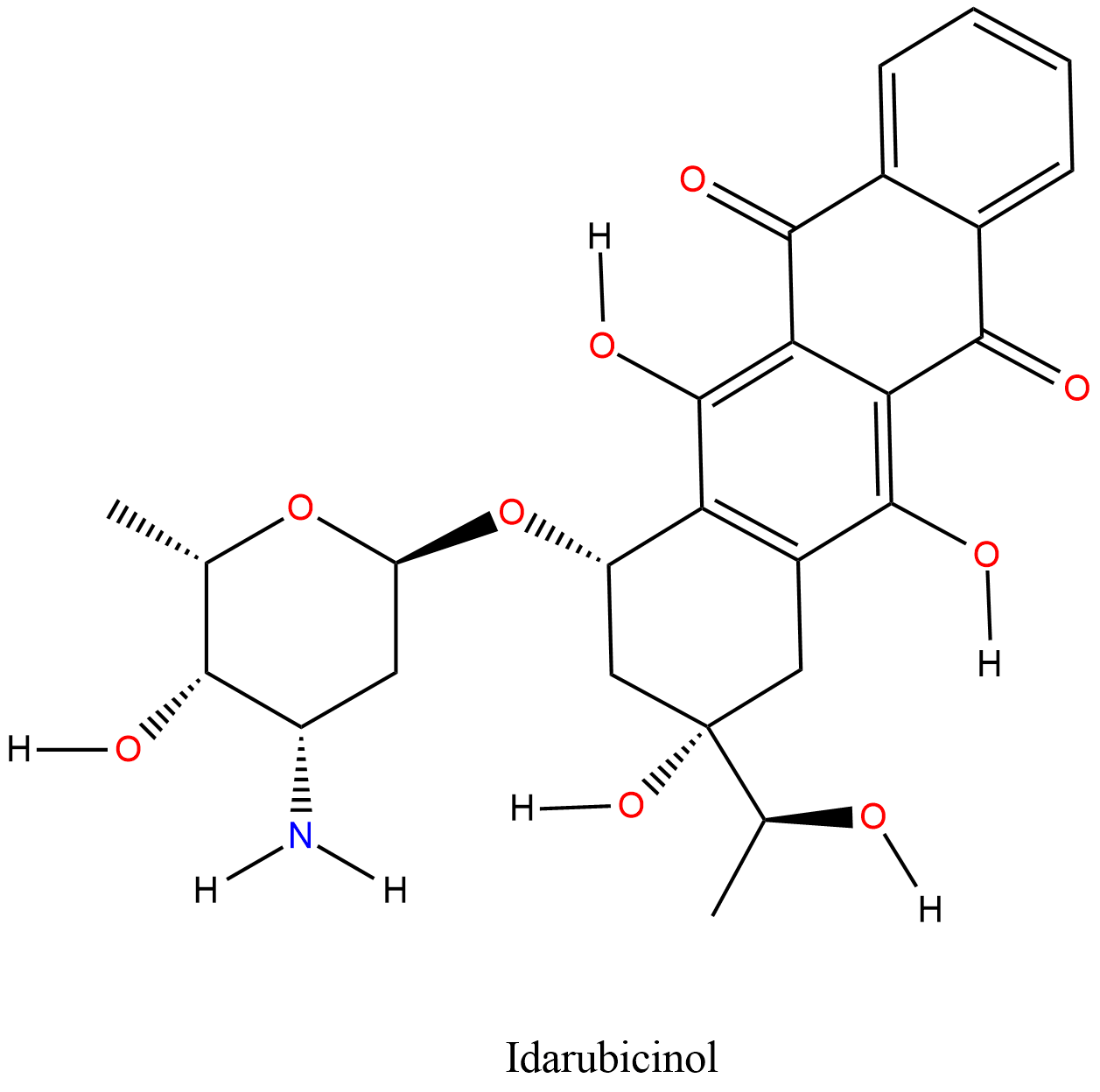


**j**

**k**

**l**

**g**

**Figure S1.** The 12 anthracycline molecules. First column shows the pristine forms, second column shows the metabolites, and the third column shows the salt cation forms. (a to c) Doxorubicin, (d to f) Epirubicin, (g to i) Daunorubicin, and (j to l) Idarubicin.

**Figure S2.** Thermodynamic factors of all systems as a function of time in ns. Pressure in bar (light blue), Temperature in Kelvin (dark blue), Total energy in KJ/mol (light green), Volume in nm^3^ (dark green). Top: Myocardial membrane. Top to bottom: Doxorubicin, Epirubicin, Daunorubicin, and Idarubicin. Left to right: pristine forms, salt forms, and metabolite forms.


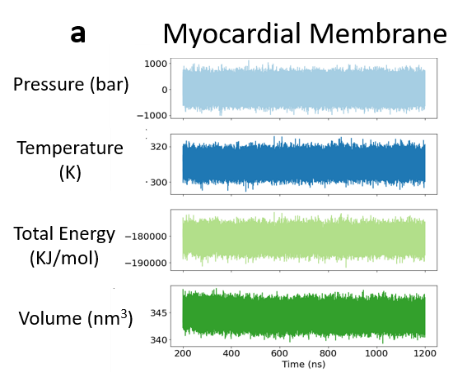

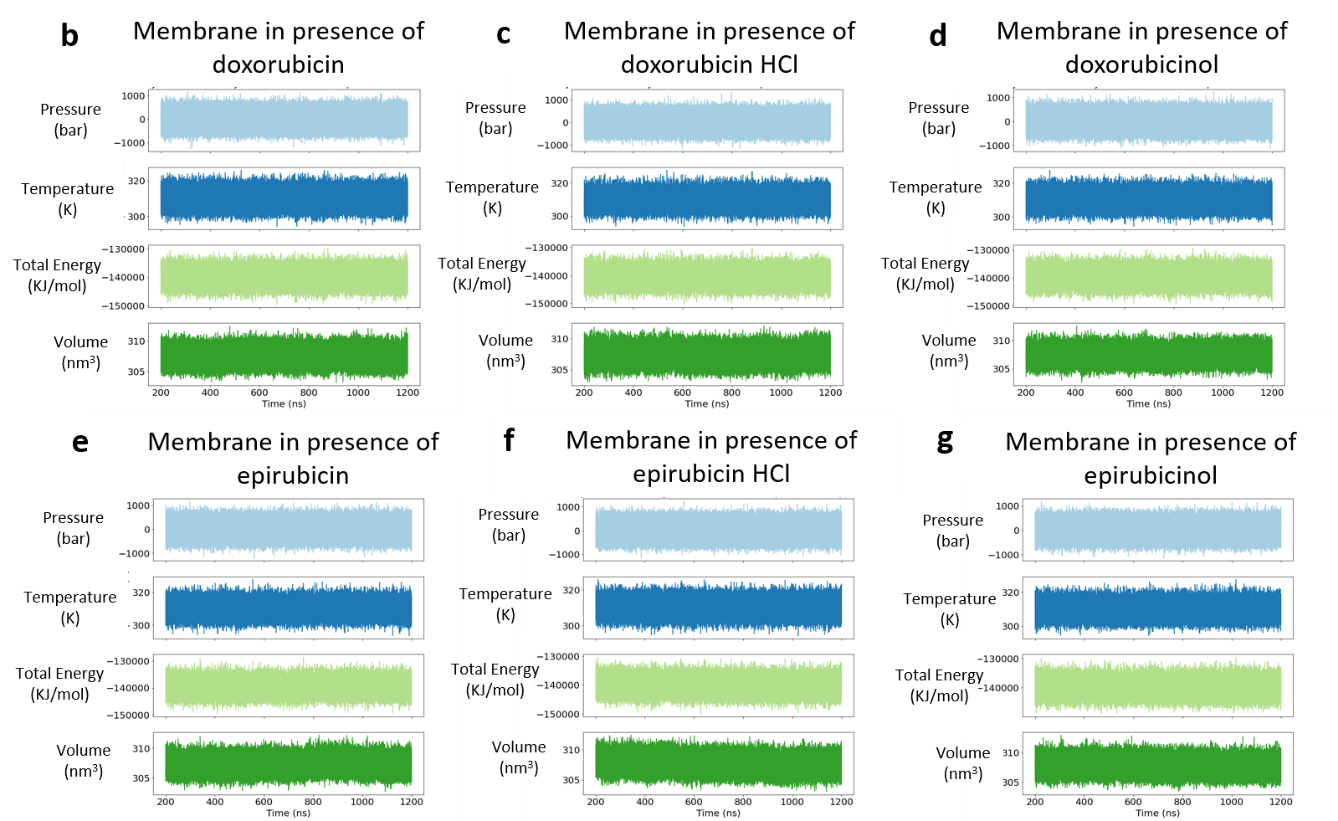

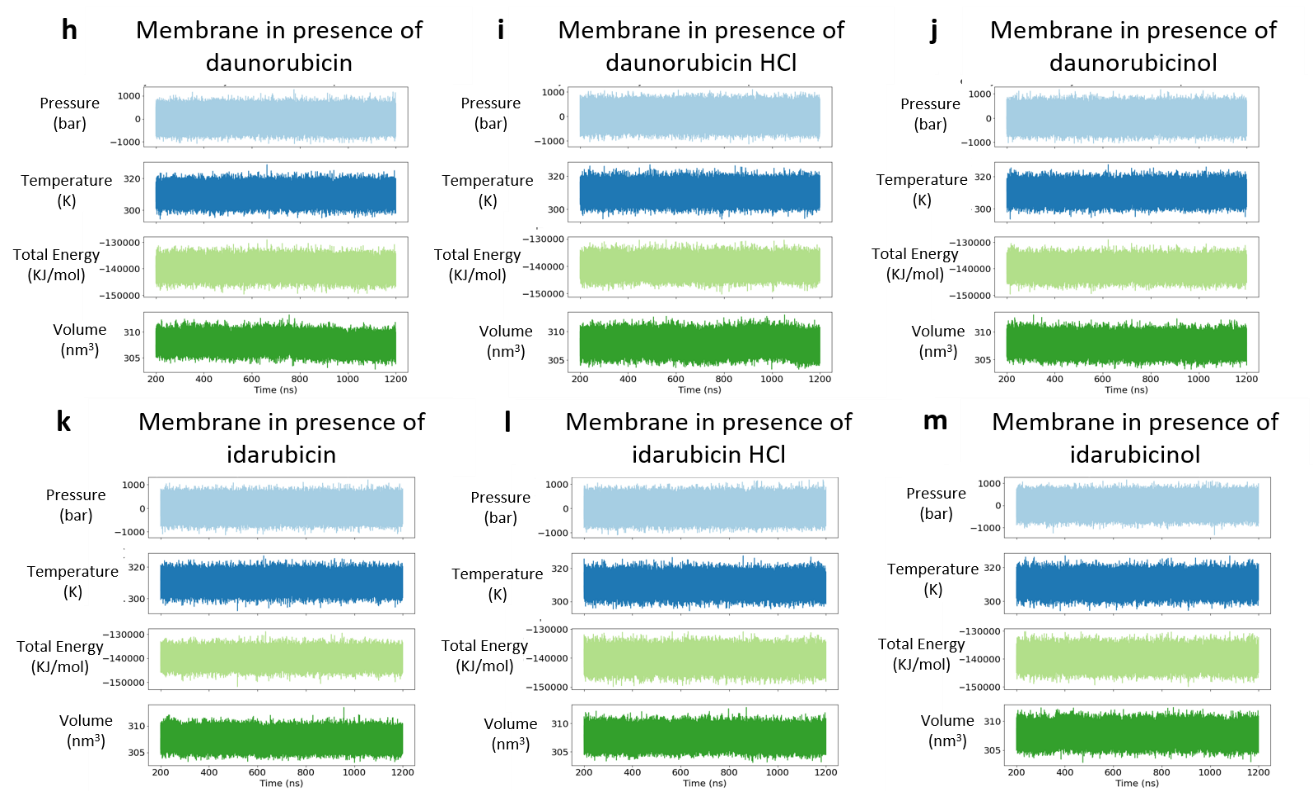


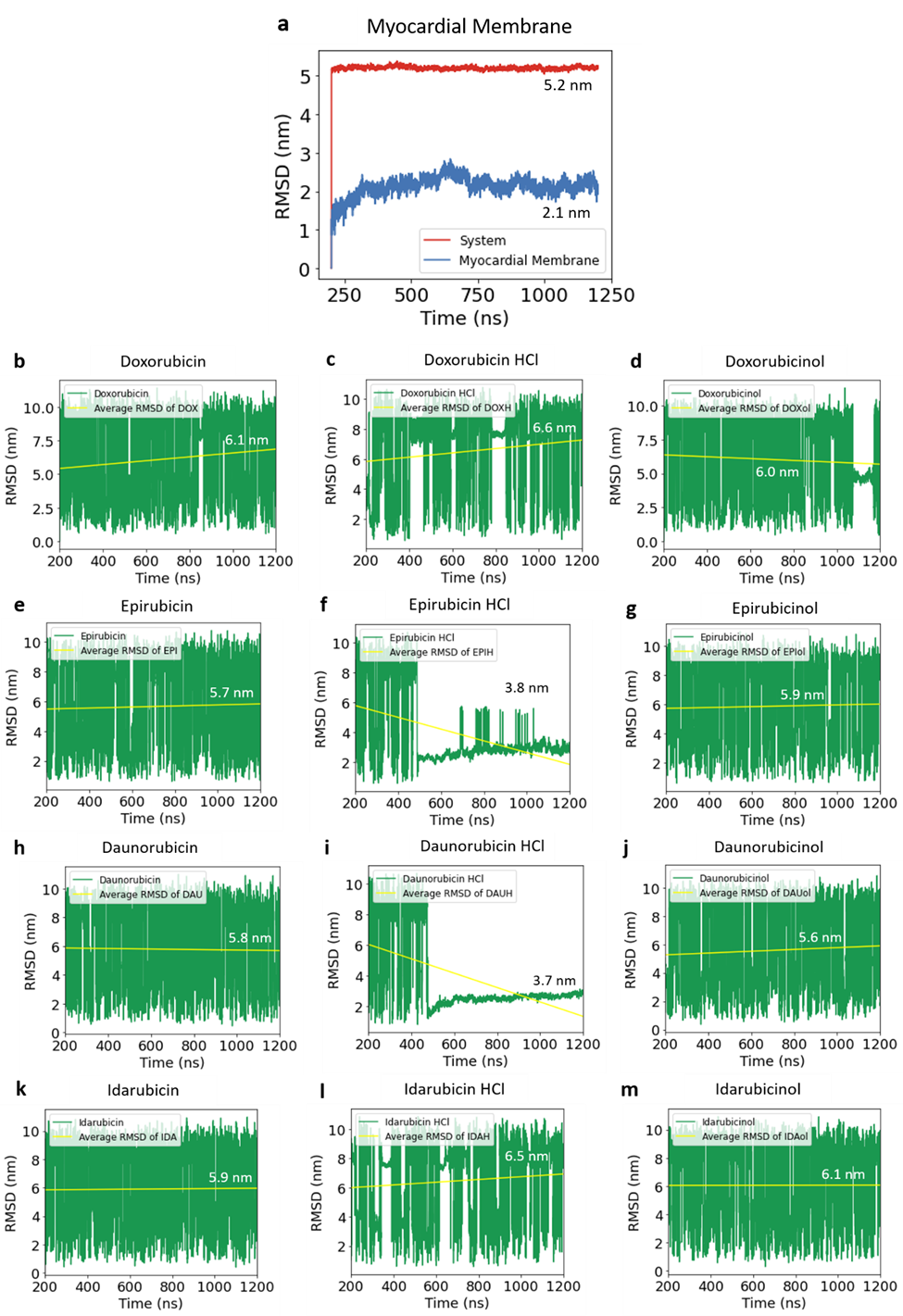


**Figure S3.** Root mean square deviation, RMSD, of all systems. Whole system (red), myocardial membrane (blue), drug molecules (green), average RMSD of drug molecules (yellow, numerical value denoted on each graph). Top: Myocardial membrane. Top to bottom: Doxorubicin, Epirubicin, Daunorubicin, and Idarubicin. Left to right: pristine forms, salt forms, and metabolite forms.


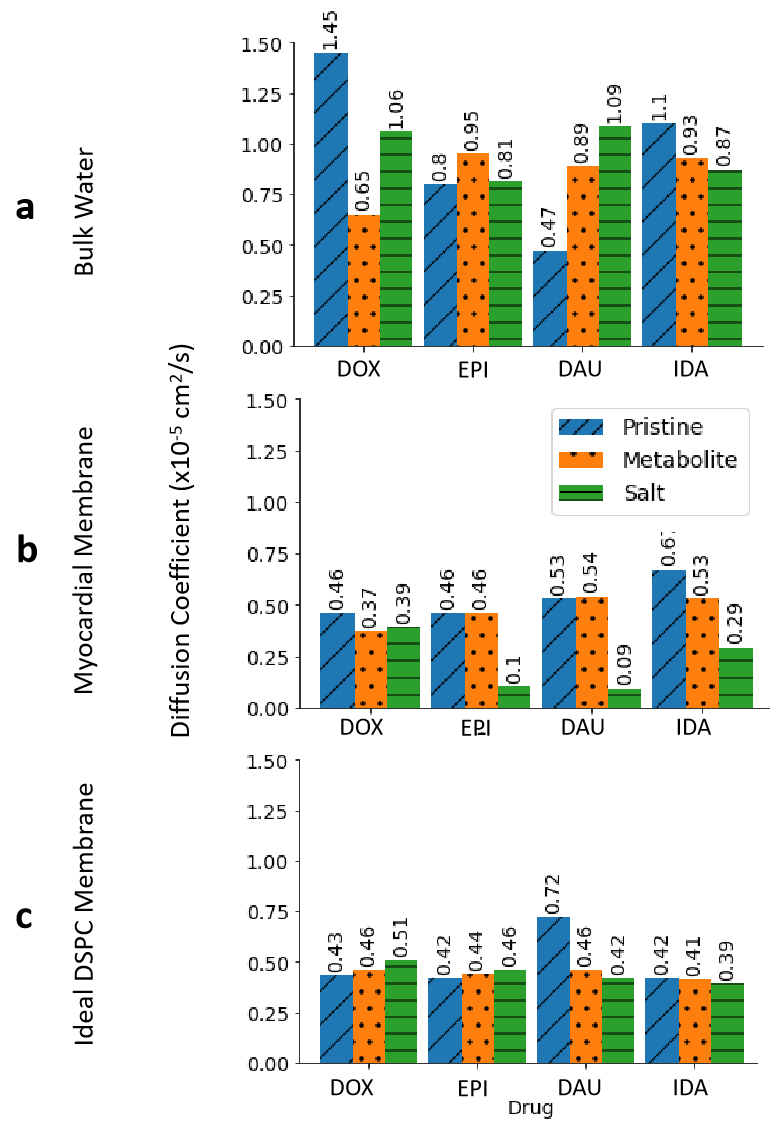


**Figure S4.** Diffusion Coefficient (x10^-5^ cm^2^/s) of each molecule (a) in bulk water, (b) near the myocardial membrane, and (c) near the ideal DSPC membrane as calculated by our molecular dynamics simulations.


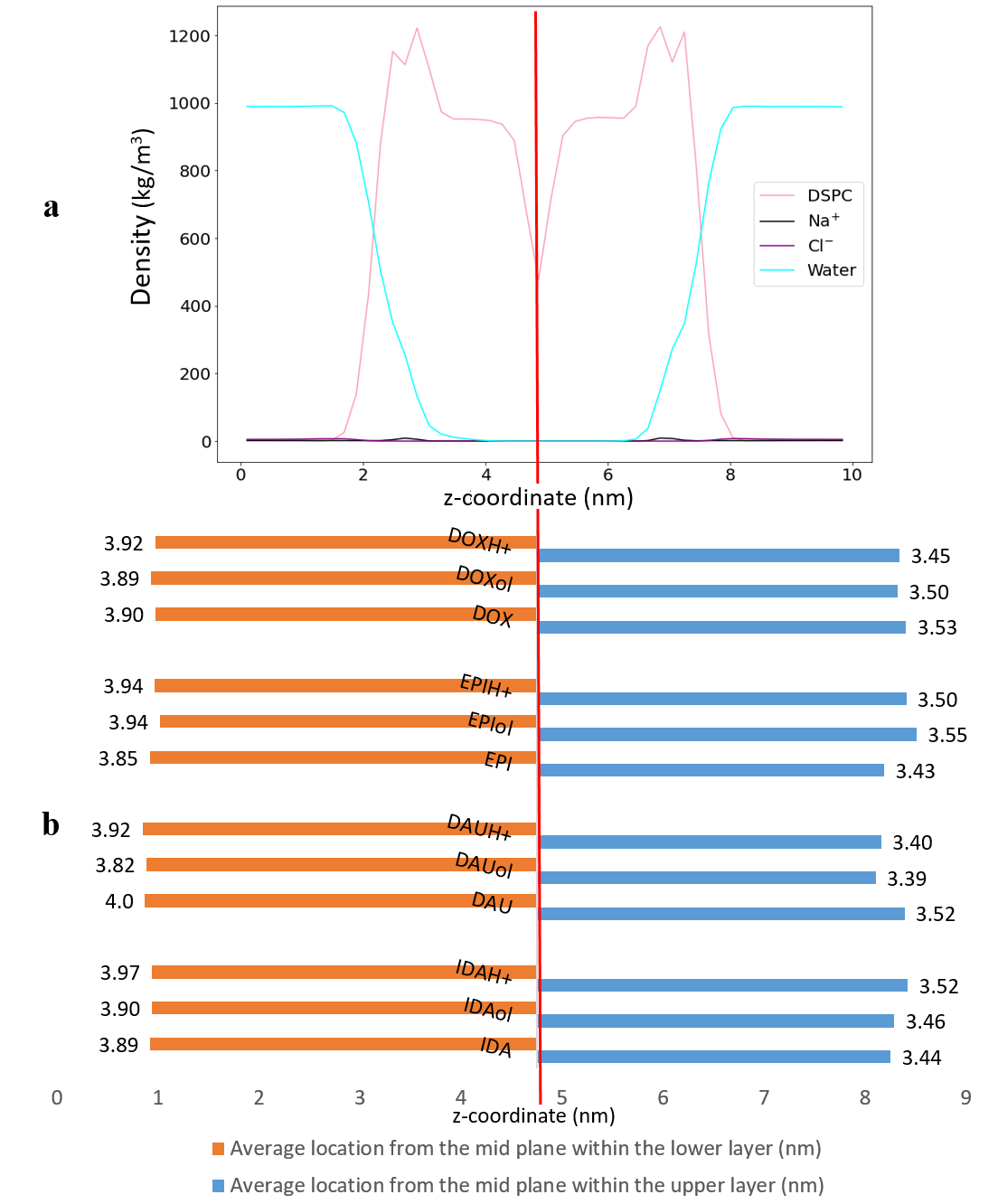


**Figure S5.** (a) The one dimensional density profile of the ideal DSPC membrane in (kg/m^3^) and (b) a graphical representation of the average location in (nm) of each anthracycline molecule from the mid plane of the DSPC membrane with the upper layer (blue bars) and the lower layer (orange bars). The edge of the bars show the location of the molecules with respect to the mid plane of the ideal DSPC membrane. The numerical annotation is the distance in (nm) between the molecules’ average locations and the mid plane. The vertical red line in panels (a) and (b) represents the mid plane.

**
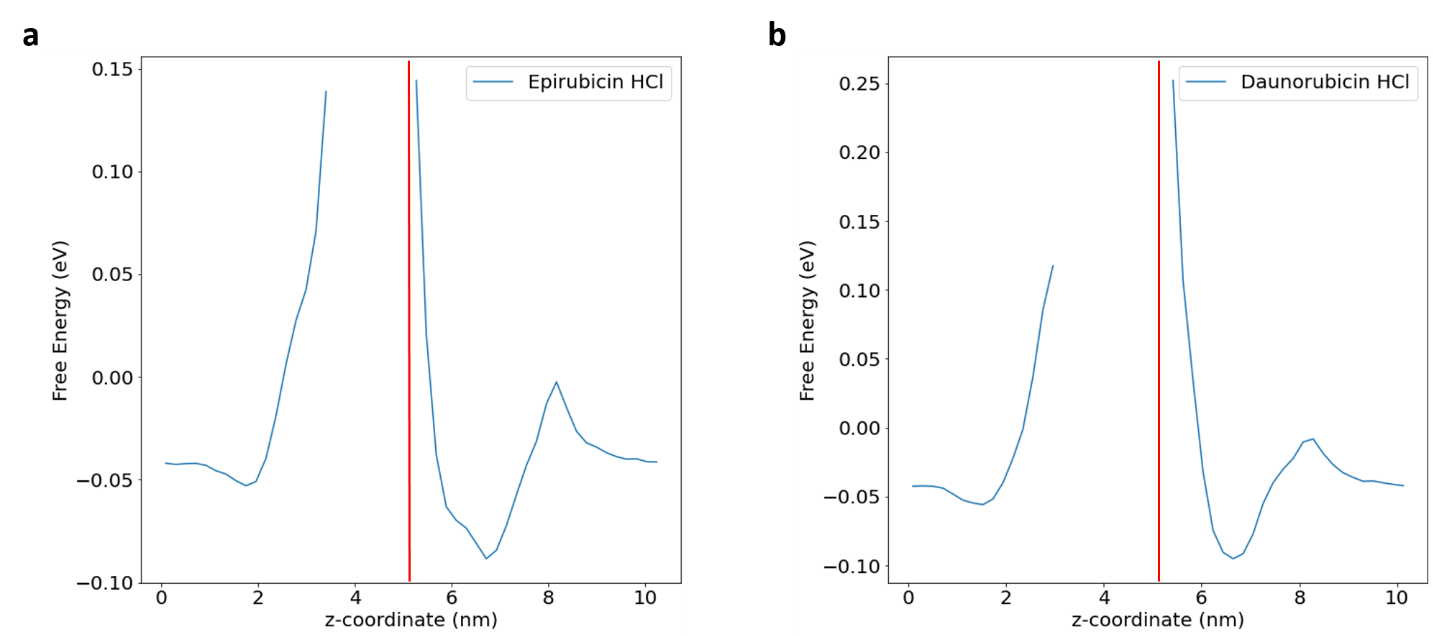
**

**Figure S6.** Free energy profiles of (a) Epirubicin HCl and (b) Daunorubicin HCl. A red line represents the mid plane and separates both profiles into two sides: left is the lower layer and right is the upper layer of the myocardial membrane. The cutout peaks are the energy barriers of the lipid tails. They are cutout because they were not captured during these simulations duration. The second smaller peaks in the upper layer are the energy barriers of the headgroups that the molecules were able to permeate during the 1.2 μs simulation time. It is expected that there should be a third peak associated with the activation barrier of crossing the head groups of the lower layer. However, since these two molecules spent most of the simulation time near or within the upper layer, accurate sampling of the third activation barrier of the polar groups of the lower layer was not achieved in our simulations. The free energy profiles presented here are calculated from the natural logarithm of the density profiles modulated by the factor –k_B_T, where T is the temperature in kelvin and k_B_ is Boltzmann constant[1].

**Table S1.** Radius of gyration (R_g_) in nm of anthracylcine molecules in bulk water and in proximity to the myocardial membrane.

| Drug molecule | Average radius of gyration (R_g_) | |
| --- | --- | --- |
|  | In bulk water | In proximity to myocardial membrane |
| Doxorubicin | 0.48 | 0.48 |
| Doxorubicinol | 0.48 | 0.48 |
| Doxorubicin HCl | 0.47 | 0.47 |
| Epirubicin | 0.48 | 0.48 |
| Epirubicinol | 0.48 | 0.48 |
| Epirubicin HCl | 0.48 | 0.48 |
| Daunorubicin | 0.47 | 0.47 |
| Daunorubicinol | 0.47 | 0.47 |
| Daunorubicin HCl | 0.47 | 0.47 |
| Idarubicin | 0.46 | 0.46 |
| Idarubicinol | 0.46 | 0.46 |
| Idarubicin HCl | 0.45 | 0.45 |

**Table S2.** Residence time (in % of total simulation duration) that each anthracycline molecule spends near each phospholipid of the myocardial membrane at a cut-off distance of 0.3 nm.

| Drug Molecule | % of time the drug spends near each phospholipid at a distance of less than or equal to 0.3nm | | |
| --- | --- | --- | --- |
|  | Doxorubicin Family - Upper Layer | | |
|  | DSPC | DSPE | SSM |
| Doxorubicin | 10.78% | 3.52% | 2.76% |
| Doxorubicinol | 11.48% | 3.32% | 3.76 |
| Doxorubicin HCl | 8.80% | 3.70% | 2.76% |
|  | Doxorubicin Family - Lower Layer | | |
|  | DSPC | DSPE | DSPS |
| Doxorubicin | 8.88% | 1.78% | 1.06% |
| Doxorubicinol | 16.10% | 11.20% | 3.38% |
| Doxorubicin HCl | 29.35% | 18.78% | 15.30% |
|  | Epirubicin Family - Upper Layer | | |
|  | DSPC | DSPE | SSM |
| Epirubicin | 9.92% | 2.64% | 2.72% |
| Epirubicinol | 12.22% | 3.04% | 3.94% |
| Epirubicin HCl | 73.27% | 71.45% | 9.18% |
|  | Epirubicin Family - Lower Layer | | |
|  | DSPC | DSPE | DSPS |
| Epirubicin | 9.70% | 3.02% | 2.94% |
| Epirubicinol | 10.88% | 3.90% | 2.04% |
| Epirubicin HCl | 40.07% | 22.0% | 2.86% |
|  | Daunorubicin Family - Upper Layer | | |
|  | DSPC | DSPE | SSM |
| Daunorubicin | 8.54% | 2.40% | 2.80% |
| Daunorubicinol | 12.94% | 4.22% | 3.38% |
| Daunorubicin HCl | 73.75% | 69.49% | 72.51% |
|  | Daunorubicin Family - Lower Layer | | |
|  | DSPC | DSPE | DSPS |
| Daunorubicin | 7.46% | 2.14% | 1.62% |
| Daunorubicinol | 7.86% | 1.98% | 1.44% |
| Daunorubicin HCl | 5.92% | 2.28% | 2.34% |
|  | Idarubicin Family - Upper Layer | | |
|  | DSPC | DSPE | SSM |
| Idarubicin | 6.76% | 1.38% | 1.80% |
| Idarubicinol | 10.36% | 1.96% | 2.76% |
| Idarubicin HCl | 9.92% | 4.56% | 2.82% |
|  | Idarubicin Family - Lower Layer | | |
|  | DSPC | DSPE | DSPS |
| Idarubicin | 7.14% | 2.48% | 2.10% |
| Idarubicinol | 10% | 3.22% | 1.86% |
| Idarubicin HCl | 26.65% | 12.56% | 17.58% |

**Table S3.** The average number of hydrogen bonds between each anthracycline molecule and each phospholipid of the myocardial membrane*.*

| Drug Molecule | Average Number of Hydrogen Bonds between Anthracycline Molecules and each Phospholipid | | |
| --- | --- | --- | --- |
|  | Doxorubicin Family - Upper Layer | | |
|  | DSPC | DSPE | SSM |
| Doxorubicin | 0.008 | 0.009 | 0.002 |
| Doxorubicinol | 0.01 | 0.01 | 0.003 |
| Doxorubicin HCl | 0.04 | 0.02 | 0.0006 |
|  | Doxorubicin Family - Lower Layer | | |
|  | DSPC | DSPE | DSPS |
| Doxorubicin | 0.006 | 0.006 | 0.005 |
| Doxorubicinol | 0.02 | 0.02 | 0.02 |
| Doxorubicin HCl | 0.13 | 0.13 | 0.13 |
|  | Epirubicin Family - Lower Layer | | |
|  | DSPC | DSPE | DSPS |
| Epirubicin | 0.007 | 0.010 | 0.0008 |
| Epirubicinol | 0.03 | 0.011 | 0.009 |
| Epirubicin HCl | 0.86 | 0.56 | 0.04 |
|  | Epirubicin Family - Lower Layer | | |
|  | DSPC | DSPE | DSPS |
| Epirubicin | 0.019 | 0.009 | 0.013 |
| Epirubicinol | 0.021 | 0.018 | 0.015 |
| Epirubicin HCl | 0.04 | 0.015 | 0.03 |
|  | Daunorubicin Family - Upper Layer | | |
|  | DSPC | DSPE | SSM |
| Daunorubicin | 0.008 | 0.006 | 0.00017 |
| Daunorubicinol | 0.01 | 0.013 | 0.003 |
| Daunorubicin HCl | 0.51 | 0.32 | 0.046 |
|  | Daunorubicin Family - Lower Layer | | |
|  | DSPC | DSPE | DSPS |
| Daunorubicin | 0.0020 | 0.0058 | 0.0048 |
| Daunorubicinol | 0.002 | 0.003 | 0.0055 |
| Daunorubicin HCl | 0.016 | 0.0088 | 0.024 |
|  | Idarubicin Family - Upper Layer | | |
|  | DSPC | DSPE | SSM |
| Idarubicin | 0.0023 | 0.0042 | 0.0017 |
| Idarubicinol | 0.009 | 0.008 | 0.002 |
| Idarubicin HCl | 0.040 | 0.018 | 0.0043 |
|  | Idarubicin Family - Lower Layer | | |
|  | DSPC | DSPE | DSPS |
| Idarubicin | 0.0042 | 0.0082 | 0.010 |
| Idarubicinol | 0.006 | 0.009 | 0.007 |
| Idarubicin HCl | 0.14 | 0.089 | 0.19 |

**Table S4.** The average number of hydrogen bonds between each anthracyline molecule and water. These were calculated from the 1.2 μs simulations for the molecules interacting with the myocardial membrane.

| Drug molecule | Average Number of Hydrogen Bonds between Anthracycline Molecules and Water |
| --- | --- |
| Doxorubicin | 11.67 ± 2.11 |
| Doxorubicinol | 12.40 ± 2.25 |
| Doxorubicin HCl | 10.85 ± 2.19 |
| Epirubicin | 11.63 ± 2.12 |
| Epirubicinol | 12.51 ± 2.21 |
| Epirubicin HCl | 7.12 ± 3.17 |
| Daunorubicin | 10.31 ± 1.96 |
| Daunorubicinol | 11.15 ± 2.03 |
| Daunorubicin HCl | 5.56 ± 3.40 |
| Idarubicin | 9.30 ± 1.92 |
| Idarubicinol | 10.12 ± 1.95 |
| Idarubicin HCl | 8.59 ± 2.08 |

**Table S5.** Comparison between the experimentally determined diffusion coefficient values from literature and the calculated values from our MD simulations.

|  | Diffusion Coefficient of Pristine Anthracyclines (×10^-5^ cm^2^/s) | |
| --- | --- | --- |
|  | Experimentally determined values in aqueous solutions[2]–[4] | Our computationally calculated values in bulk water |
| DOX | 1.5 | 1.45 |
| EPI | 0.504 | 0.8 |
| IDA | 0.75 | 1.1 |

**Table S6.** The diffusion coefficient of each molecule in all dimensions near the myocardial membrane and in bulk water.

| Drug molecule | Diffusion Coefficient (x10^-5^ cm^2^/s) | | | | |
| --- | --- | --- | --- | --- | --- |
|  | Near the Myocardial Membrane | | | | In Bulk Water |
|  | Average in all dimensions | X | Y | Z | Average in all dimensions |
| Doxorubicin | 0.46 | 0.68 | 0.67 | 0.02 | 1.5 |
| Doxorubicinol | 0.37 | 0.59 | 0.50 | 0.02 | 0.65 |
| Doxorubicin HCl | 0.39 | 0.49 | 0.63 | 0.04 | 1.1 |
|  | Epirubicin Family | | | | |
| Epirubicin | 0.46 | 0.66 | 0.69 | 0.01 | 0.80 |
| Epirubicinol | 0.46 | 0.81 | 0.55 | 0.02 | 0.95 |
| Epirubicin HCl | 0.10 | 0.08 | 0.19 | 0.02 | 0.81 |
|  | Daunorubicin Family | | | | |
| Daunorubicin | 0.54 | 0.82 | 0.77 | 0.02 | 0.47 |
| Daunorubicinol | 0.54 | 0.71 | 0.89 | 0.02 | 0.89 |
| Daunorubicin HCl | 0.09 | 0.15 | 0.12 | 0.01 | 1.09 |
|  | Idarubicin Family | | | | |
| Idarubicin | 0.67 | 1.1 | 0.85 | 0.007 | 1.1 |
| Idarubicinol | 0.53 | 0.80 | 0.78 | 0.01 | 0.93 |
| Idarubicin HCl | 0.29 | 0.47 | 0.37 | 0.04 | 0.87 |

**Table S7.** The dipole moment calculations for each molecule using force field (GROMACS) and density functional theory (DFT).

| Dipole Moment (Debye) | | | |
| --- | --- | --- | --- |
| Software and Calculation Details  Drug | GROMACS | | CRYSTAL17 |
|  | Single Precision | Double Precision | *Gas Phase Optimization* |
|  | after 100ns MD simulation in bulk water | after minimization in  Vacuum | M06-2X functional  &  cc-PVDZ basis set |
|  | Energy Step | |  |
|  | 1 etmol | 0.1 etmol |  |
| Doxorubicin | 6.99 | 7.78 | 2.56 |
| Epirubicin | 3.90 | 8.50 | 5.18 |
| Daunorubicin | 6.12 | 5.78 | 6.87 |
| Idarubicin | 5.25 | 4.50 | 2.47 |
| Doxorubicinol | 6.12 | 3.48 | 6.84 |
| Epirubicinol | 5.84 | 5.72 | 3.96 |
| Daunorubicinol | 11.22 | 8.17 | 3.86 |
| Idarubicinol | 7.61 | 6.46 | 3.98 |

**Movies S1 and S2**

**Movie S1.** (a) EPIH^+^ (Epirubicin HCl salt) partially permeating the upper layer of the myocardial membrane model versus (b) EPIH+ freely moving in proximity of the ideal DSPC membrane model.

**Movie S2.** (a) DAUH^+^ (Daunorubicin HCl salt) partially permeating the upper layer of the myocardial membrane model versus (b) DAUH+ freely moving in proximity of the ideal DSPC membrane model.

**SI References**

[1] S. Kerisit and S. C. Parker, “Free Energy of Adsorption of Water and Metal Ions on the {101̄4} Calcite Surface,” *J. Am. Chem. Soc.*, vol. 126, no. 32, pp. 10152–10161, Aug. 2004, doi: 10.1021/ja0487776.

[2] E. V. Shil’ko, I. V. Dudkin, A. Yu. Smolin, K. V. Krukovskii, and A. I. Lotkov, “Estimation of the Diffusion Coefficient of Doxorubicin Molecules in a Water Solution in the Volume of a Porous Carrier Medium,” *Russ Phys J*, vol. 62, no. 12, pp. 2319–2323, Apr. 2020, doi: 10.1007/s11182-020-01983-y.

[3] R. Hajian, E. Ekhlasi, and R. Daneshvar, “Spectroscopic and Electrochemical Studies on the Interaction of Epirubicin with Fish Sperm DNA,” *E-Journal of Chemistry*, vol. 9, no. 3, pp. 1587–1598, Jan. 2012, doi: 10.1155/2012/738678.

[4] H. Eda Satana Kara, “Redox mechanism of anticancer drug idarubicin and in-situ evaluation of interaction with DNA using an electrochemical biosensor,” *Bioelectrochemistry*, vol. 99, pp. 17–23, Oct. 2014, doi: 10.1016/j.bioelechem.2014.06.002.
